# Thermodynamically-guided machine learning modelling for predicting the glass-forming ability of bulk metallic glasses

**DOI:** 10.1038/s41598-022-15981-2

**Published:** 2022-07-11

**Authors:** Alireza Ghorbani, Amirhossein Askari, Mehdi Malekan, Mahmoud Nili-Ahmadabadi

**Affiliations:** 1 School of Metallurgy and Materials Engineering, College of Engineering, University of Tehran, Tehran, 11155-4563 Iran; 2 Computer Engineering Department, Amirkabir University of Technology, Tehran, 15916-34311 Iran

**Keywords:** Engineering, Materials science

## Abstract

Glass-forming ability (GFA) of bulk metallic glasses (BMGs) is a determinant parameter which has been significantly studied. GFA improvements could be achieved through trial-and-error experiments, as a tedious work, or by using developed predicting tools. Machine-Learning (ML) has been used as a promising method to predict the properties of BMGs by removing the barriers in the way of its alloy design. This article aims to develop a ML-based method for predicting the maximum critical diameter (D_max_) of BMGs as a factor of their glass-forming ability. The main result is that the random forest method can be used as a sustainable model (*R*^*2*^ = 92%) for predicting glass-forming ability. Also, adding characteristic temperatures to the model will increase the accuracy and efficiency of the developed model. Comparing the measured and predicted values of D_max_ for a set of newly developed BMGs indicated that the model is reliable and can be truly used for predicting the GFA of BMGs.

## Introduction

Bulk Metallic Glasses (BMGs) are metallic alloys with high strength, good corrosion resistance, good hardness, and good wear resistance because of their amorphous structure^1^. The excellent properties of BMGs make them a good choice for applications like cardiovascular stents, micro fuel cells (MFC), wear-resistant gears, and catalysts^2^. However, designing BMGs is challenging due to their non-equilibrium nature related to the metastability of amorphous structures^3^. Moreover, the low glass-forming ability of BMGs has limited their application^4^. Therefore, understanding the glass-forming ability of BMGs is crucial in alloy design. The critical casting diameter (D_max_) has been used as a parameter of the glass-forming ability (GFA) of BMGs^5^. The glass-forming ability of BMGs has a relationship with some of the thermodynamic properties, such as characteristic temperatures, i.e., the glass transition temperature, T_g_, the onset crystallization temperature, T_x,_ and the liquidus temperature, T_l_. The characteristic temperatures of BMGs are strongly affected by the composition of these alloys^6–10^.

Scientists tried to establish a relation between characteristic temperatures and glass-forming ability^6,11–14^. Inoue^15^ used the difference between T_x_ and T_g_ (i.e., ΔT_x_ expression) to evaluate the glass-forming ability of BMGs. Lu and Liu^13^ propose that using γ ($=\frac{{T}_{x}}{{T}_{g}+{T}_{l}}$) could guide scientists to compare the glass-forming ability of alloy systems. Tripathi et al.^16^, combining the thermodynamics and principles of genetic programming, developed a new parameter (i.e., the G_p_ criterion) to measure the glass-forming ability of BMGs. It has been suggested that the stability of the liquid phase and the glass’s resistance to crystallization should be considered simultaneously to increase the GFA of alloys^17^. Therefore, it is impossible to use some of these parameters as indicators of GFA. For example, the ΔT_x_ parameter only considers the stability of the glassy phase and not the ease of glass formation. Similarly, T_rg_ (= T_g_/T_l_) is a parameter that only considers the ease of glass formation^18^. In addition, although developing different parameters able scientists to evaluate and predict the glass-forming ability of alloy systems, most of these expressions have a low correlation with the glass-forming ability and will fail in predicting the D_max_ of newly developed alloy systems^16,19^.

In recent years, ML has been known as one of the best routes in predicting the glass-forming ability of BMGs^20–23^. Scientists have used ML as a promising route for materials design and discovery^24^. The ML is the science of developing models that will become more efficient over time and is going to replace old methods of finding a solution to relate input features to output features with new attitudes to solving the problem with artificial intelligence^25^. By using ML modeling, scientists are enable to relate characteristic features of material to its properties^26^. Therefore, the data-driven approach is compelling in accelerating the exploration of new metallic glasses^27^.

Recently, Ward et al.^28^ built a Random Forest model on a dataset of 6315 alloys for predicting three critical parameters of BMGs, such as critical casting diameter or D_max_. Using different ML methods, they found that decision tree-based methods, especially random forest, are the most accurate methods for investigating the properties of BMGs. These researchers used 201 features categorized in seven different groups, including stoichiometric, elemental property statistics, valence shell, ionicity, proximity to crystalline compounds, cluster packing efficiency, and probability of glass-formation attributes, to build a model for predicting the D_max_ as a criterion of GFA. The feature space of this model was massive, so it needed much larger amounts of data than other published models, and the training time was tedious. As a result, the correlation coefficient of this model was lower for the BMG dataset, comparing the original dataset consisting BMGs and ribbon-forming glasses.

Xiong et al.^11^ trained seven different models for predicting the GFA of BMGs. These researchers selected four different parameters from a feature space of 11 basic elemental properties, including metallic radius (R_m_), the heat of fusion (H_f_), melting point (T_m_), and specific heat capacity (C_m_), to assess the maximum critical diameters of BMGs. This research depicts that the random forest method is suitable for assessing the properties of BMGs. Although the number of features was significantly lower comparing the model built by Ward et al., the accuracy was still around 80%. Also, computing the value of selected features was sophisticated and needed more thermodynamic data.

Deng and Zhang^29^ used Random Forest to train a model for predicting the glass-forming ability of metallic alloys. They input parameters such as Total Electronegativity (TEN), atomic size difference (δ), average atomic volume, and the mixing entropy (S_m_) to the random forest model to predict the glass-forming ability of BMGs. Although this research was very inspiring, calculating the input parameters of these models is challenging, and the accuracy was not efficient in some cases. Considering the published researches, it is indicated that developing a model using fewer attributes and a larger dataset is required, and this model could be used to predict the GFA of BMGs in an efficient method.

This article used the Random Forest method with Python 3.9 to train a model based on parameters derived from characteristic temperatures relations for predicting the critical casting diameter (D_max_) of BMGs, using a database of 715 different alloys consisting of Zr-based, Mg-based, Cu-based, La-based, Fe-based and other BMGs. In this research, the fivefold cross-validation has been used to improve the accuracy of the model. The fivefold cross-validation randomly divided the dataset into 572 alloys for use as training and 143 alloys as test sets. Then, the characteristic temperatures were added as input features to guide the model thermodynamically.

## Methodology

**Figure 1:**
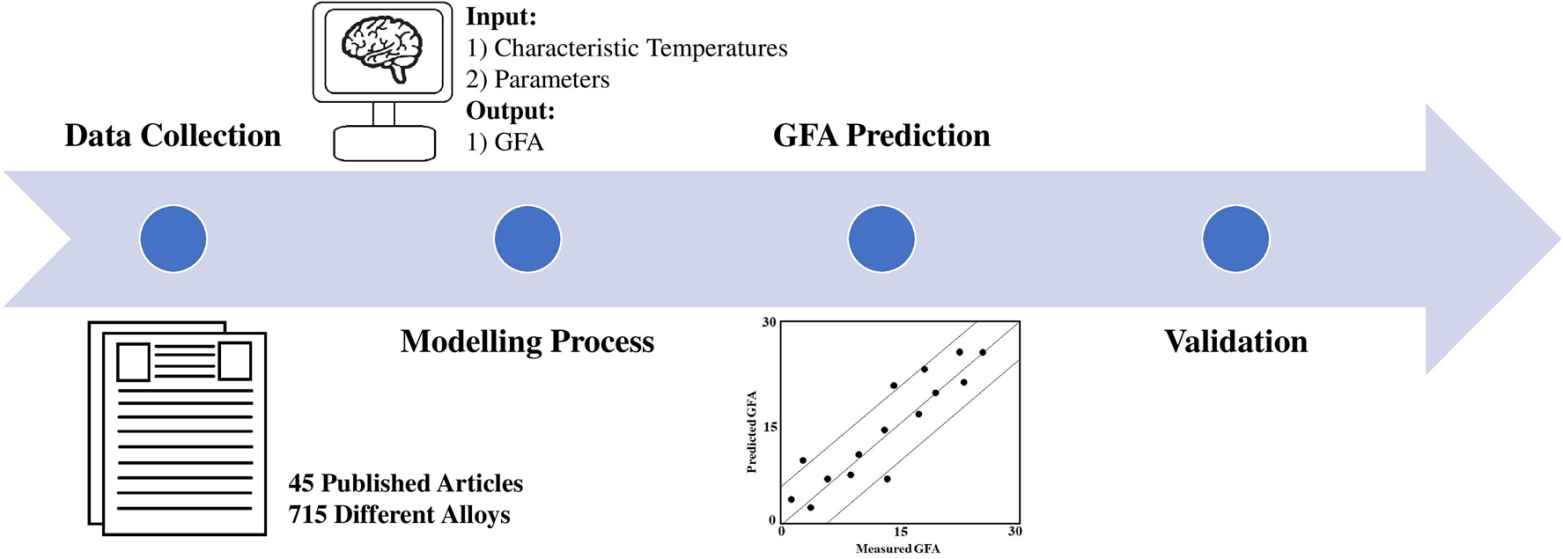
Schematic demonstration of steps involved in the process of ML modeling.

The whole process of modeling consists of four steps. The schematic demonstration of these steps is represented in Fig. 1. The very first step of modeling is collecting a suitable dataset. The next step is processing the dataset with input features to attain the desired output. It then can be followed by representing results. The final step is the validation of the model.

### Data collection

In the ML, it is essential to use a suitable dataset. Due to the purpose of ML and attaining a reliable result from modeling, the dataset used in this article was collected from 44 different published articles^12,19,30–71^, including 715 different BMGs. Furthermore, 17 different parameters were used as input features to predict the maximum critical diameter (D_max_) of BMGs. It is noticeable that all parameters can be calculated using characteristic temperatures. Although liquid fragility is another important parameter that has a straightforward relationship with the GFA, researchers need to have the relaxation time of metallic glasses to measure this parameter. In most papers used in the dataset, there is insufficient information for determining this parameter. Besides that, since one of the parameters we used as an input feature (i.e., the reduced glass-transition temperature or T_rg_) is essential in determining the liquid fragility parameter, adding the liquid fragility parameter will not benefit the model’s accuracy^72^. Therefore, the liquid fragility parameter has not been used in this research. The parameters including the ideal values are represented in Table 1. When the parameter’s value tends to this ideal value, the GFA (i.e., D_max_) of BMGs is increased.

**Table 1:** Parameters (GFA criteria) expressed by characterization temperatures used as input features in this article (* means the ideal value has not been measured for this parameter).

No.	Parameter	Ideal value	Year established	References
1	${T}_{rg}=\frac{{T}_{g}}{{T}_{l}}$	1.0	1969	^73^
2	$\Delta {T}_{x}={T}_{x}-{T}_{g}$	0.0	1995	^15^
3	$\alpha =\frac{{T}_{x}}{{T}_{l}}$	1.0	2005	^74^
4	$\beta =\frac{{T}_{x}}{{T}_{g}}+\frac{{T}_{g}}{{T}_{l}}$	2.0	2005	^74^
5	$new \beta =\frac{{T}_{x}\times {T}_{g}}{({T}_{l}-{T}_{x}{)}^{2}}$	ꝏ	2008	^75^
6	$\gamma =\frac{{T}_{x}}{{T}_{g}+{T}_{l}}$	0.5	2002	^13,76^
7	${\gamma }_{m}=\frac{2{T}_{x}-{T}_{g}}{{T}_{l}}$	1.0	2007	^77^
8	$\delta =\frac{{T}_{x}}{{T}_{l}-{T}_{g}}$	ꝏ	2006	^78^
9	$\varnothing ={T}_{rg}(\frac{{\Delta T}_{x}}{{T}_{g}}{)}^{0.143}$	0.0	2007	^79^
10	$\omega =\frac{{T}_{g}}{{T}_{x}}-\frac{2{T}_{g}}{{T}_{g}+{T}_{l}}$	0.0	2009	^33,80^
11	${\omega }_{m}=\frac{{2{T}_{x}-T}_{g}}{{T}_{x}+{T}_{l}}$	0.0	2015	^81^
12	$\theta =\frac{({T}_{x}+{T}_{g})}{{T}_{l}}\times [\frac{({T}_{x}-{T}_{g})}{{T}_{l}}{]}^{0.0728}$	0.0	2009	^82^
13	$\upxi =\frac{\Delta {T}_{x}}{{T}_{x}}+\frac{{T}_{g}}{{T}_{l}}$	1.0	2008	^83^
14	${\beta }^{^{\prime}}=\frac{{T}_{g}}{{T}_{x}}-\frac{{T}_{g}}{{1.3\times T}_{l}}$	*	2011	^6^
15	$\Delta {T}_{rg}=\frac{{T}_{x}-{T}_{g}}{{T}_{l}-{T}_{g}}$	*	2004	^84^
16	${G}_{p}=\frac{{T}_{g}({T}_{x}-{T}_{g})}{({T}_{l}-{T}_{X}{)}^{2}}$	*	2016	^16^
17	${\gamma }_{c}=\frac{{3T}_{x}-{2T}_{g}}{{T}_{l}}$	1.0	2010	^85^

### Modelling process

At the first step of modeling, all of the values of the features normalized with the Min–Max method to fall in the [0, 1] domain^86^. Normalization is a preprocessing step in ML modeling. It has been shown that normalization can help scientists establish more accurate models. Model fitting and model learning are affected by variables measured at different scales and could result in decreased efficiency of the model. The typical way to deal with this potential problem is to perform feature-wise normalization, such as Min–Max scaling, before fitting a model^87^. The formula of the Min–Max method is represented in Eq. (1):

1 $${X}_{new}=\frac{x-\mathrm{min}(x)}{\mathrm{max}\left(x\right)-\mathrm{min}(x)}$$

where the X_new_ is the normalized value of x and max(x) and min(x) are maximum and minimum values of feature x, respectively.

Recursive Feature Elimination (RFE) is used with the Random Forest algorithm for feature selection. The RFE starts with pruning the least essential feature from the set of features and is repeated on the pruned set until the desired number of features to select is eventually reached^88^. Then, three characteristic temperatures were added to the model with the highest correlation value and all features. In the following step, fivefold cross-validation is used to split the dataset into five groups. Four groups form the training set, and one performs as a testing set. The whole process of modeling was repeated 100 times. The mean of all modeling was used to ensure the accuracy and generalization ability of the model^89^. Then, the squared correlation coefficient (R^2^) and mean absolute error (MAE) were selected to evaluate the accuracy and generalization ability of the RF model. Equations (2) and (3) represent the formula of these methods:

2 $${R}^{2}=\frac{(n\sum_{i=1}^{n}f\left({x}_{i}\right){y}_{i}-\sum_{i=1}^{n}f({x}_{i})\sum_{i=1}^{n}{y}_{i}{)}^{2}}{(n\sum_{i=1}^{n}f({x}_{i}{)}^{2}-(\sum_{i=1}^{n}f\left({x}_{i}\right){)}^{2}-(n\sum_{i=1}^{n}{y}_{i}^{2}-(\sum_{i=1}^{n}{y}_{i}{)}^{2})}$$

3 $$MAE=\frac{1}{n}\sum_{i=1}^{n}\left|f\left({x}_{i}\right)-{y}_{i}\right|$$

where n is the number of samples and f(x_i_) and y_i_ represent the predicted and experimental values of the i_th_ sample, respectively.

### Validation

For validation of the model, the characteristic temperatures of the four alloys studied in the previous work^90^ were used as inputs. The predicted D_max_ of these alloys was compared with the measured values of D_max_. In order to evaluate the effect of adding characteristic temperatures to the model, the overfitting and squared correlations of the initial models and models with characteristic temperatures were compared.

## Results and discussion

### Feature selection

**Figure 2:**
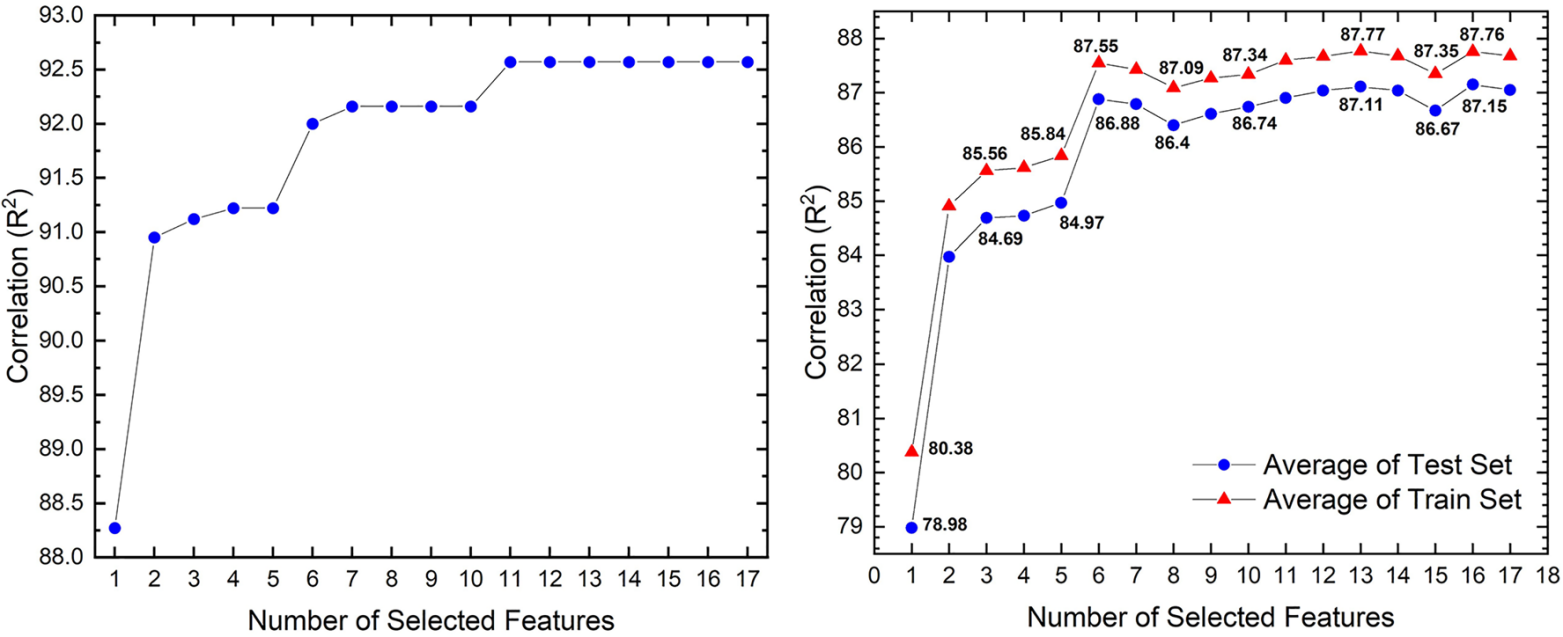
The squared correlation coefficient (R^2^) of 5-fold cross-validated models based on the number of selected features for (**a**) test set of the most accurate models with the highest R^2^ and (**b**) averages train sets and test sets of 100 repeated models.

The Random Forest Regression (RFR) with 5-fold cross-validation was conducted on the BMGs dataset 100 times to have a reliable model. Then, the Recursive Feature Elimination (RFE) algorithm was used to determine the squared correlation coefficient (R^2^) of models with the different number of selected features, as shown in Fig. 2. It can be seen from Fig. 2a that the correlation of the best model with a certain number of selected features (i.e., the highest correlation for the model with the specific number of selected features) can be divided into three different parts, where the correlation will maintain the same level at that part. Moreover, it is indicated that models with more than 11 selected features have the highest correlation. In fact, this figure indicates that when we select just one parameter (from 17 parameters) and train the model, the testset accuracy of the best model would be around 88.25%. Similarly, when we choose two parameters and train the model, the accuracy will increase to approximately 91%. This increase will continue until we select 11 parameters. At this step, the accuracy would be around 92.5%. After that, the change in accuracy would be imperceptible when we increase the number of selected parameters in training the model. Figure 2b represents the average coefficient of all 100 repeated modeling and illustrates that the model with 13 selected features has the highest average correlation value compared to other models. Although the model with 16 features also had a correlation coefficient near the model with 13 features, it is preferable to have the least number of features^91^. Moreover, overfitting is higher in the models with more parameters^92^. As a result, the model including 13 features was selected as the optimal model for further research in this article.

To ensure that the characteristic temperatures will not be eliminated if we use RFE for all parameters, including characteristic temperatures, we conducted RFE on a model with all 20 parameters. The selected parameters, in this case, were the same as the model with 13 features + 3 characteristic temperatures, i.e., T_g_, T_x,_ and T_l_, and the characteristic temperatures were selected as features.

**Figure 3:**
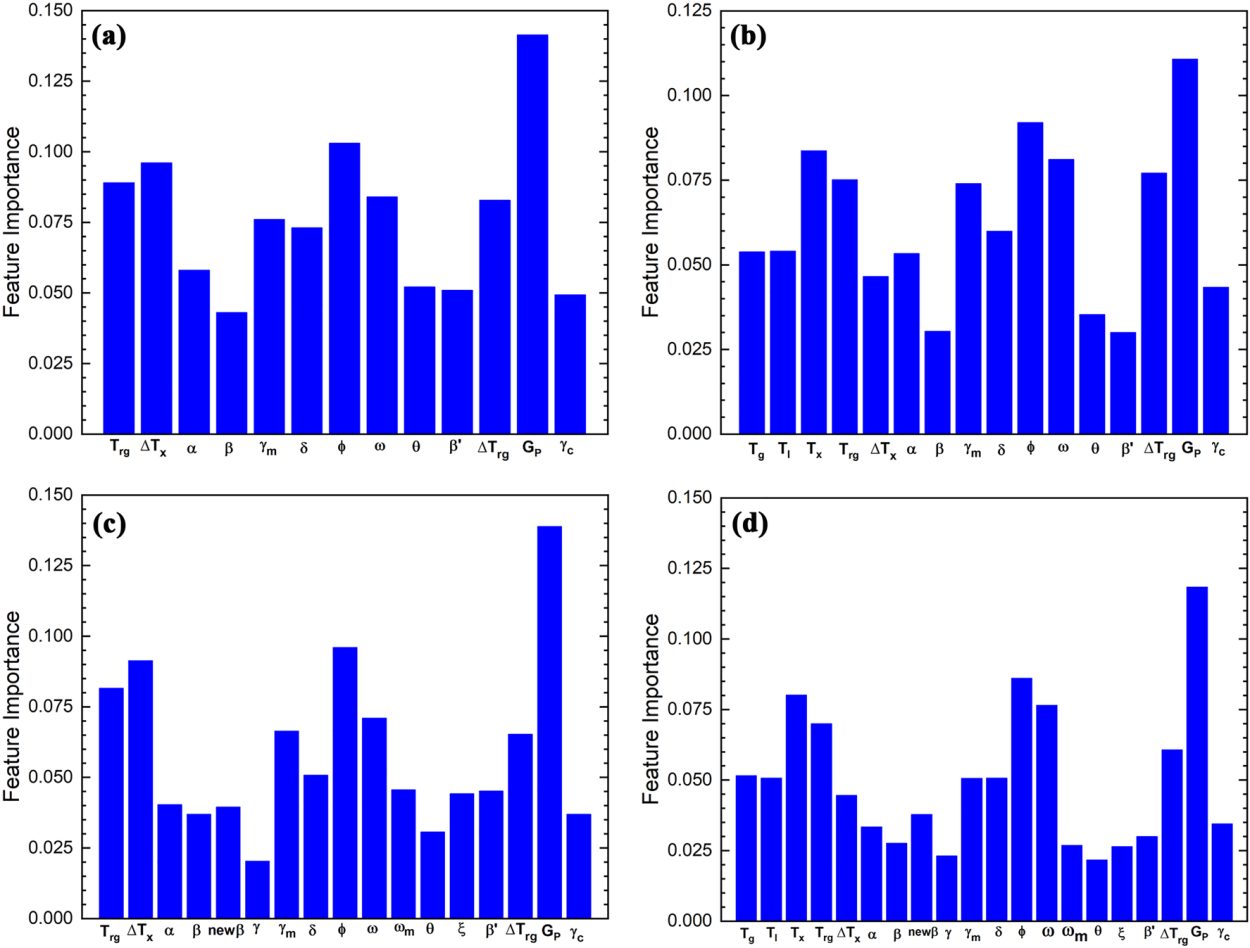
The feature importance of the model with (**a**) 13 selected features, (**b**) 13 selected features and added characteristic temperatures, (**c**) 17 selected features, and (**d**) 17 selected features and added characteristic temperatures.

To evaluate the effect of adding characteristic temperatures to the model and thermodynamically guiding the model, T_g_, T_x,_ and T_l_ were added to the optimal model (i.e., the model with 13 features) and the model with all features. To evaluate every single feature’s importance in modeling, the feature importance algorithm is represented in Fig. 3.

Figure 3a,b are related to the feature importance of models with 13 different features without and with characteristic temperatures. Also, Fig. 3c,d are for models with all parameters without and with characteristic temperatures. It is evident that in all of the modeling, the parameter of G_p_ is the most crucial feature. There are some reasons why this parameter is so important, and actually, it has the highest importance in all of the modelings. First, the Gp expression has been extracted using genetic programming. Scientists could generate several different solutions using this method and choose the most reliable solution^93^. Secondly, the expression itself can be divided into two different parts that are shown in Eq. (4):

4 $${G}_{P}=\frac{{T}_{g}({T}_{x}-{T}_{g})}{({T}_{l}-{T}_{x}{)}^{2}}=\frac{{T}_{g}}{{T}_{l}-{T}_{x}}\times \frac{{T}_{x}-{T}_{g}}{{T}_{l}-{T}_{x}}$$

Increasing Tg in the first part of Eq. (4) will increase the viscosity, and the glass-formation process will facilitate. Also, T_l_–T_x_ area is prone to crystallization, so minimizing this part of the Equation is desirable^75^. Besides, it is optimal to have an extended supercooled liquid area (i.e., ${T}_{x}-{T}_{g}$) in BMGs as crystallization will not occur at this area^78^. On the other hand, the G_p_ expression proposes that high T_x_ will increase liquid stability and lead to better glass-forming ability. Based on Wakasugi et al.^94^, the viscosity of supercooled liquid will increase with increasing the ratio of T_x_/T_l,_ and increasing the viscosity of supercooled liquid will lead to high glass-forming ability. In conclusion, the G_p_ expression inherits the phenomenological attributes of glass-forming ability in BMGs and shows a good correlation with D_max_^16^.

### GFA prediction

**Figure 4:**
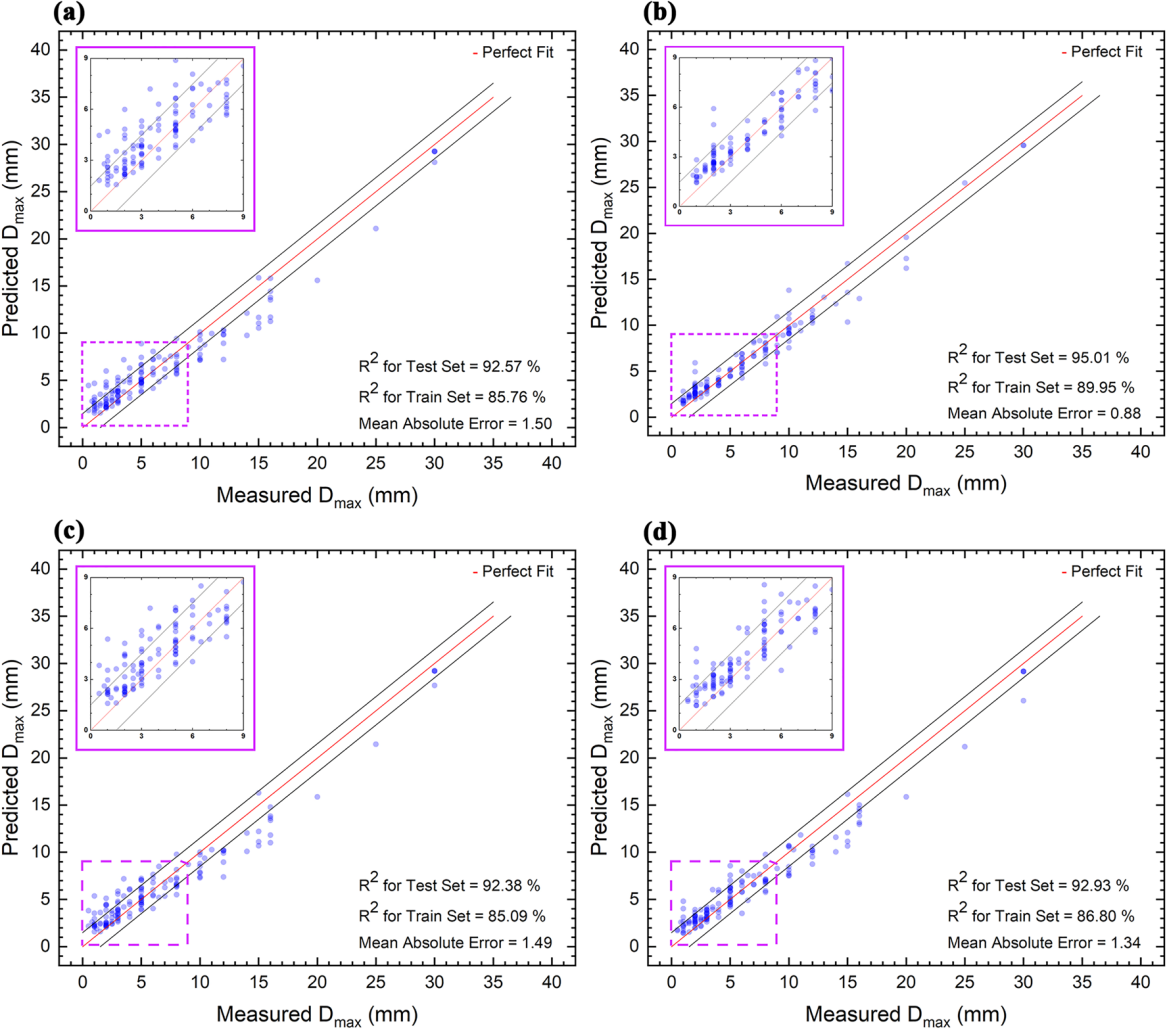
The predicted D_max_ values against measured D_max_ for the model with (**a**) 13 selected features, (**b**) 13 selected features and added characteristic temperatures, (**c**) 17 selected features, and (**d**) 17 selected features and added characteristic temperatures.

Figure 4 shows the testing set’s predicted Dmax versus the measured D_max_. As can be seen, adding characteristic temperatures to the models increases the squared correlation coefficient for the train set and test set and decreases the mean absolute error. The best result is for the model with 13 features and added characteristic temperatures. Our model had two different outliers, which were Pd_40_Cu_30_Ni_10_P_20_ with a D_max_ of 72 mm^95^, and Zr_41.2_Ti_13.8_Cu_12.5_Ni_10_Be_22.5_ with a D_max_ of 50 mm^76^. These two alloys are outliers because the dataset does not have any other alloy with a high GFA like these alloys^28,96^. The model with 13 features and added characteristic temperatures is the most accurate, with a R^2^ of 95.01% and a MAE of 0.88 mm.

### The effect of characteristic temperatures on GFA prediction

**Figure 5:**
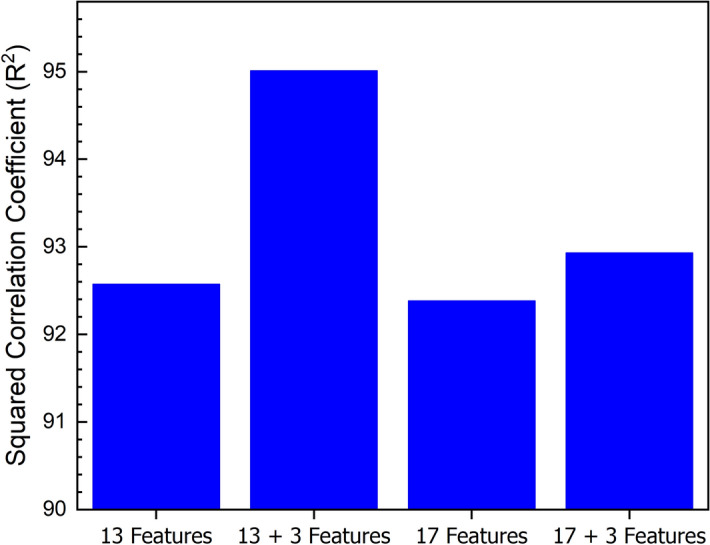
The squared correlation coefficient for models with 13 features with and without characteristic temperatures and 17 features with and without characteristic temperatures.

Figure 5 indicates the correlation coefficient for models without characteristic temperatures and those including characteristic temperatures. The R^2^ of the test set increased by adding characteristic temperatures in both models, i.e., the model with 13 features and the model with 17 features. It is important to note that mean absolute error was reduced by introducing characteristic temperatures. Characteristic temperatures and selected parameters have a synergic effect on each other because; by using these parameters, the thermodynamic approach of the model will improve. Firstly, all the input parameters are dimensionless relations, including the characteristic temperatures. It is expected that introducing the individual characteristic temperatures will increase the weight of these crucial parameters, which in turn leads the model to be more accurate. In an ideal glassy system with high glass-forming ability, the glass transition temperature (and crystallization temperature) should be very high and the liquidus temperature very low^3^. Secondly, the two critical criteria for high glass-forming ability are the liquid phase’s stability and crystallization resistance^76^. Although in most of the parameters, these two factors have been considered, some of them, such as T_rg_ and ΔT_x,_ are only based on the stability of the liquid phase^15,73^. It has been indicated that the stability in the equilibrium state and in the undercooled state depends on the liquidus and glass transition temperatures, respectively. On the other hand, resistance to crystallization is a parameter that has a linear relationship with the glass transition temperature^13,76^. As a result, by introducing the characteristic temperatures as input parameters, ignorance of parameters like ease of glass formation and resistance to crystallization will be compensated.

**Figure 6:**
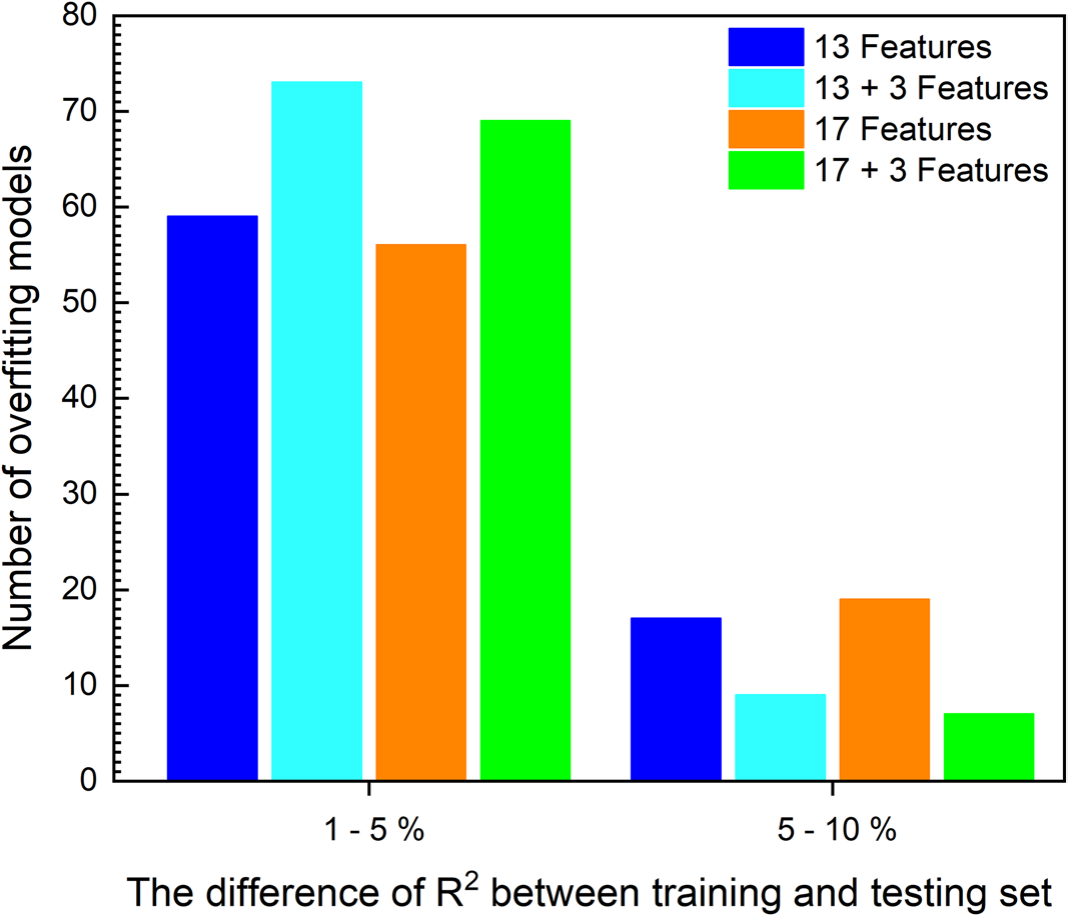
The number of overfitting ML models with/without characteristic temperatures.

Overfitting is one of the most critical problems in ML modeling that cause us to have a low coefficient correlation in the testing set while having a very well-fitted model on the training set. The Overfitting/Underfitting can be determined using the squared correlation coefficient of the training and test sets^97^. For evaluating the effect of the introduction of characteristic temperatures on the overfitting problem^24^, the number of models with a difference of R^2^ value between the training set and testing set in the range of 1–5% and 5–10% are plotted respectively as shown in Fig. 6. The overfitting problem and this difference are dependent on each other. The overfitting problem is more severe in cases where the difference of R^2^ value is higher^98^. It is evident that adding the characteristic temperatures to the models reduces the occurrence of overfitting and improves the efficiency and accuracy of the model.

### Validation

In order to validate the established model (the one including 13 features and characteristic temperatures), the measured D_max_ as the glass-forming ability of four different alloys along with the related data were extracted from reference^90^. The data of alloys entered into the model, and then the predicted D_max_ is compared with the experimental results, as shown in Table 2. The modeling results were very close to the experimental results, as shown by low discrepancy between the experimental and predicted results, which is in the range of regression value (Fig. 4b). This works shows that neither 13 dimensionless parameters proposed by different researchers nor three characteristic temperatures proposed in this work could predict the GFA accurately. However, when three characteristic temperatures plus 13 parameters were employed by ML modeling, a rather more accurate result was depicted. In fact, most of the GFA results in literature have been obtained by trial-and-error experiments without sufficient scientific background, e.g., thermodynamic analysis. This matter is mostly due to non-equilibrium and complicated feathers of solidification in BMG alloys. The successful introduction of three characteristic temperatures, which are closely related to the thermodynamics of BMG alloys, bears in mind that more scientific exploring on the composition-structure-properties of BMG could be very informative to provide enough knowledge for predicting GFA to lessen too many experimental studies.

**Table 2:** Experimental validation of ML model using the four different alloys.

Alloy composition	Glass transition temp	Crystallization temp	Liquidus temp	Measured critical diameter (from Ref^90^)	Predicted critical diameter (this work)	Measured and predicted discrepancy%	Refs.
Cu_50_Zr_43_Al_7_	713.0	781.0	1205.0	10.0	10.8	+ 8	^90^
(Cu_50_Zr_43_Al_7_)_98_Y_2_	696.0	770.0	1165.0	15.0	14.3	− 4.7	
(Cu_50_Zr_43_Al_7_)_96_Y_4_	679.0	715.0	1150.0	12.0	11.3	− 6	
(Cu_50_Zr_43_Al_7_)_94_Y_6_	665.0	703.0	1145.0	8.0	8.0	0.0	

## Conclusion

In summary, this work conducted a random forest model combined with Recursive Feature Elimination to predict the D_max_ (GFA) of BMGs on a dataset of 714 different alloys. The results showed that the model with 13 selected features has the highest coefficient correlation value (R^2^ = 92%) and is very efficient. The feature importance algorithm showed that the G_p_ parameter is the most crucial parameter in all modeling. Adding characteristic temperatures, i.e., T_g_, T_x,_ and T_l_, to the models will improve the accuracy (R^2^ up to 95%) and efficiency of models, resulting in overcoming the overfitting problem. Comparing the measured and predicted values of D_max_ for a set of newly developed BMGs indicated that the model is reliable and can be used for predicting the GFA of BMGs.

## Supplementary Information

Supplementary Information 1.

Supplementary Information 2.

## Data Availability

The collected dataset is represented in “Supplementary Information Files”. Developed codes for Machine-Learning are accessible through http://github.com/naha7789/GFA-predicting.
