# Supplementary material for: Thermodynamically-guided machine learning modelling for predicting the glass-forming ability of bulk metallic glasses
Source: Sci Rep. 2022 Jul 11;12:11754. doi: 10.1038/s41598-022-15981-2 (PMC9273633; doi:10.1038/s41598-022-15981-2)
Supplement: Supplementary file 1 — Supplementary Information 1. [file 41598_2022_15981_MOESM1_ESM.docx]

**Characteristic Temperature of BMGs Dataset**

| **Alloy Composition** | **T_g_**  **(K)** | **T_x_**  **(K)** | **T_l_**  **(K)** | **D_max_** | **Ref.** |
| --- | --- | --- | --- | --- | --- |
| Cu60Zr33Ti7 | 740.0 | 768.0 | 1191.0 | 3.0 | ^1^ |
| Cu54Ag6Zr33Ti7 | 709.0 | 738.0 | 1135.0 | 6.0 |  |
| Cu46.4Ag11.6Zr35Ti7 | 689.0 | 732.0 | 1119.0 | 6.0 |  |
| Cu44.25Ag14.75Zr35Ti6 | 693.0 | 730.0 | 1112.0 | 8.0 |  |
| Cu44.25Ag14.75Zr36Ti5 | 700.0 | 734.0 | 1115.0 | 10.0 |  |
| Cu49Hf42Al9 | 778.0 | 863.0 | 1249.0 | 10.0 |  |
| Cu47Zr11Ni8Ti34 | 671.0 | 717.0 | 1160.0 | 3.0 | ^2^ |
| Cu60Zr30Ti10 | 713.0 | 750.0 | 1151.0 | 4.0 |  |
| Cu60Hf25Ti15 | 730.0 | 795.0 | 1160.0 | 4.0 |  |
| (Cu60Zr30Ti10)98Y2 | 707.0 | 757.0 | 1122.0 | 5.0 |  |
| (Cu60Zr30Ti10)90Be10 | 720.0 | 762.0 | 1130.0 | 5.0 |  |
| (Cu60Zr30Ti10)99Sn1 | 730.0 | 776.0 | 1155.0 | 5.0 |  |
| Cu55Ag5Zr30Ti10 | 704.0 | 733.0 | 1149.0 | 3.0 | ^3^ |
| Cu50Ag10Zr30Ti10 | 694.0 | 726.0 | 1130.0 | 4.0 |  |
| Cu45Ag15Zr30Ti10 | 687.0 | 717.0 | 1121.0 | 5.0 |  |
| Cu40Ag20Zr30Ti10 | 677.0 | 708.0 | 1125.0 | 3.0 |  |
| Cu35Ag25Zr30Ti10 | 677.0 | 706.0 | 1138.0 | 2.0 |  |
| Cu45Ni5Ag10Zr30Ti10 | 710.0 | 738.0 | 1160.0 | 5.0 |  |
| Cu60Hf20Ti20 | 740.0 | 767.0 | 1211.0 | 4.0 |  |
| Cu60Hf17.5Ti22.5 | 732.0 | 755.0 | 1229.0 | 3.0 |  |
| Cu42Zr42Ag8Al8 | 715.0 | 779.0 | 1218.0 | 10.0 | ^4^ |
| Cu40Zr44Ag8Al8 | 711.0 | 789.0 | 1180.0 | 15.0 |  |
| Cu38Zr46Ag8Al8 | 705.0 | 793.0 | 1145.0 | 15.0 |  |
| Cu36Zr48Ag8Al8 | 690.0 | 791.0 | 1143.0 | 15.0 |  |
| Cu34Zr50Ag8Al8 | 695.0 | 778.0 | 1147.0 | 10.0 |  |
| Cu46Zr46Al4Ag4 | 686.0 | 767.0 | 1169.0 | 7.0 | ^5^ |
| Cu47Zr45Al4Ag4 | 688.0 | 770.0 | 1159.0 | 10.0 |  |
| Cu48Zr44Al4Ag4 | 692.0 | 779.0 | 1156.0 | 10.0 |  |
| Cu49Zr43Al4Ag4 | 694.0 | 780.0 | 1158.0 | 10.0 |  |
| Cu50Zr42Al4Ag4 | 703.0 | 784.0 | 1167.0 | 9.0 |  |
| Cu48Zr42Al6Ag4 | 711.0 | 800.0 | 1186.0 | 12.0 |  |
| Cu48Zr44Al6Ag2 | 704.0 | 788.0 | 1168.0 | 12.0 |  |
| Cu47Zr45Al5Ag3 | 702.0 | 785.0 | 1160.0 | 15.0 |  |
| Cu50Zr42Al6Ag2 | 709.0 | 802.0 | 1194.0 | 9.0 |  |
| Cu46Zr44Al4Ag6 | 692.0 | 776.0 | 1175.0 | 11.0 |  |
| Cu46Zr44Al6Ag4 | 698.0 | 778.0 | 1176.0 | 11.0 |  |
| Cu44Zr44Ag6Al6 | 698.0 | 790.0 | 1144.0 | 10.0 | ^3^ |
| Cu40Zr40Ag10Al10 | 710.0 | 765.0 | 1273.0 | 3.0 |  |
| Cu43Zr43Ag7Ti7 | 670.0 | 714.0 | 1118.0 | 5.0 |  |
| Cu43Zr43Ag7In7 | 704.0 | 748.0 | 1135.0 | 5.0 |  |
| Cu43Zr40Ag7Ti10 | 656.0 | 707.0 | 1095.0 | 7.0 |  |
| Cu64Zr36 | 787.0 | 833.0 | 1230.0 | 2.0 |  |
| Cu50Zr43Al7 | 721.0 | 792.0 | 1176.0 | 4.0 | ^6^ |
| Cu47Zr43Al7Ag3 | 716.0 | 795.0 | 1156.0 | 5.0 |  |
| Cu47Zr43Al7Be3 | 715.0 | 798.0 | 1139.0 | 6.0 |  |
| Cu43Zr43Al7Ag7 | 710.0 | 797.0 | 1125.0 | 8.0 |  |
| Cu43Zr43Al7Be7 | 710.0 | 813.0 | 1126.0 | 12.0 |  |
| Cu50Zr50 | 686.0 | 744.0 | 1237.0 | 2.0 | ^7^ |
| Cu48Zr48Ag4 | 681.0 | 743.0 | 1199.0 | 3.0 |  |
| Cu46Zr46Ag8 | 677.0 | 745.0 | 1167.0 | 4.0 |  |
| Cu45Zr45Ag10 | 683.0 | 756.0 | 1142.0 | 6.0 |  |
| Cu44Zr44Ag12 | 684.0 | 764.0 | 1156.0 | 4.0 |  |
| Cu42Zr42Ag16 | 685.0 | 757.0 | 1232.0 | 2.0 |  |
| Cu45Zr45Ag7Al3 | 688.0 | 768.0 | 1137.0 | 7.0 |  |
| Cu45Zr45Ag5Al5 | 697.0 | 783.0 | 1129.0 | 9.0 |  |
| Cu45Zr45Ag3Al7 | 708.0 | 786.0 | 1164.0 | 8.0 |  |
| Cu45Zr35Ag10Hf10 | 690.0 | 769.0 | 1171.0 | 4.0 |  |
| Cu45Zr25Ag10Hf20 | 698.0 | 783.0 | 1218.0 | 3.0 |  |
| Cu45Zr15Ag10Hf30 | 712.0 | 799.0 | 1275.0 | 2.0 |  |
| Cu45Zr5Ag10Hf40 | 720.0 | 814.0 | 1321.0 | 2.0 |  |
| Cu36Zr48Ag8Al8 | 690.0 | 791.0 | 1143.0 | 25.0 | ^8^ |
| Cu34Pd2Zr48Ag8Al8 | 699.0 | 794.0 | 1140.0 | 30.0 |  |
| Cu32Pd4Zr48Ag8Al8 | 705.0 | 795.0 | 1153.0 | 20.0 |  |
| Cu30Pd6Zr48Ag8Al8 | 709.0 | 796.0 | 1155.0 | 15.0 |  |
| Cu28Pd8Zr48Ag8Al8 | 713.0 | 798.0 | 1161.0 | 15.0 |  |
| Cu26Pd10Zr48Ag8Al8 | 720.0 | 802.0 | 1165.0 | 15.0 |  |
| Cu49Hf42Al9 | 780.0 | 847.0 | 1226.0 | 8.0 | ^9^ |
| Cu50Hf42.5Al7.5 | 780.0 | 846.0 | 1218.0 | 7.0 |  |
| Cu0.6Hf0.25Ti0.15)98Nb2 | 746.0 | 792.0 | 1184.0 | 4.0 | ^3^ |
| (Cu0.6Hf0.25Ti0.15)96Nb4 | 747.0 | 789.0 | 1188.0 | 4.0 |  |
| (Cu0.6Hf0.25Ti0.15)94Nb6 | 745.0 | 785.0 | 1190.0 | 4.0 |  |
| (Cu0.6Hf0.25Ti0.15)92Nb8 | 745.0 | 783.0 | 1198.0 | 2.5 |  |
| Cu46Zr45Al7Y2 | 693.0 | 770.0 | 1143.0 | 8.0 |  |
| Cu46Zr42Al7Y5 | 672.0 | 772.0 | 1113.0 | 10.0 |  |
| Cu49Hf42Al9 | 778.0 | 863.0 | 1249.0 | 10.0 |  |
| Cu45Zr48Al7 | 708.0 | 766.0 | 1186.0 | 5.0 |  |
| Cu47Ti33Zr11Ni8Si1 | 720.0 | 757.0 | 1157.0 | 4.0 |  |
| Cu47Ti33Zr9Nb2Ni8Si1 | 728.0 | 762.0 | 1159.0 | 5.0 |  |
| Cu47Ti33Zr7Nb4Ni8Si1 | 713.0 | 739.0 | 1172.0 | 5.0 |  |
| Cu47Ti33Zr5Nb6Ni8Si1 | 712.0 | 739.0 | 1187.0 | 2.0 |  |
| Cu47Ti33Zr3Nb8Ni8Si1 | 708.0 | 731.0 | 1228.0 | 1.0 |  |
| Cu47Ti33Nb11Ni8Si1 | 710.0 | 732.0 | 1265.0 | 0.5 |  |
| Cu57Zr36Ag7 | 712.0 | 755.0 | 1156.0 | 4.0 |  |
| Cu54Zr36Ag10Ti5 | 719.0 | 759.0 | 1146.0 | 6.0 |  |
| Cu49Zr36Ag10Ti5 | 691.0 | 737.0 | 1130.0 | 8.0 |  |
| Cu50Zr42.5Ti7.5 | 677.0 | 717.0 | 1152.0 | 5.0 |  |
| (Cu0.5Zr0.425Ti0.075)99Sn1 | 683.0 | 730.0 | 1140.0 | 6.0 |  |
| (Cu0.5Zr0.425Ti0.075)99Si1 | 683.0 | 731.0 | 1141.0 | 6.0 |  |
| (Cu0.5Zr0.425Ti0.075)98.8Sn0.6Si0.6 | 682.0 | 734.0 | 1141.0 | 7.0 |  |
| Mg80Ni10Nd10 | 454.0 | 471.0 | 878.0 | 0.6 |  |
| Mg70Ni15Nd15 | 467.0 | 489.0 | 844.0 | 1.5 |  |
| Mg75Ni15Nd10 | 450.0 | 470.0 | 790.0 | 2.8 |  |
| Mg65Cu25Er10 | 422.0 | 480.0 | 766.0 | 3.0 |  |
| Mg65Ni20Nd15 | 459.0 | 501.0 | 805.0 | 3.5 |  |
| Mg65Cu15Ag10Er10 | 427.0 | 465.0 | 733.0 | 6.0 |  |
| Mg65Cu7.5Ni7.5Zn5Ag5Y10 | 426.0 | 464.0 | 717.0 | 9.0 |  |
| Mg65Cu25Y10 | 413.0 | 473.0 | 760.0 | 4.0 |  |
| Mg65Cu25Gd10 | 423.0 | 484.0 | 740.0 | 8.0 |  |
| Mg65Cu25Dy10 | 422.0 | 492.0 | 750.0 | 3.0 |  |
| Mg65Cu25Pr10 | 413.0 | 446.0 | 784.0 | 1.0 |  |
| Mg65Cu25Nd10 | 423.0 | 456.0 | 744.0 | 1.0 |  |
| Mg65Cu25Ho10 | 417.0 | 473.0 | 751.0 | 1.0 |  |
| Mg65Cu15Ag5Pd5Gd10 | 430.0 | 472.0 | 748.0 | 10.0 |  |
| Mg65Cu25Gd5Y5 | 413.0 | 486.0 | 755.0 | 5.0 |  |
| Mg65Cu20Ni5Gd10 | 420.0 | 481.0 | 786.0 | 5.0 |  |
| Mg65Cu15Ag10Y4Gd6 | 424.0 | 467.0 | 682.0 | 8.0 |  |
| Mg65Cu15Ag10Y2Gd8 | 420.0 | 464.0 | 683.0 | 9.0 |  |
| Mg65Cu15Ag10Gd10 | 416.0 | 459.0 | 686.0 | 7.5 |  |
| Mg65Cu7.5Ni7.5Ag5Zn5Gd10 | 440.0 | 477.0 | 726.0 | 11.0 |  |
| Mg65Cu7.5Ni7.5Ag5Zn5Gd7.5Y2.5 | 438.0 | 474.0 | 719.0 | 13.0 |  |
| Mg65Cu7.5Ni7.5Ag5Zn5Gd5Y5 | 434.0 | 472.0 | 718.0 | 14.0 |  |
| Mg65Cu7.5Ni7.5Ag5Zn5Gd2.5Y7.5 | 433.0 | 473.0 | 735.0 | 9.5 |  |
| Mg65Cu7.5Ni7.5Ag5Zn5Y10 | 430.0 | 459.0 | 728.0 | 9.0 |  |
| Mg58.5Cu30.5Y11 | 422.0 | 496.0 | 762.0 | 9.0 |  |
| Mg57Cu31.5Y9.2Nd2.3 | 428.0 | 502.0 | 777.0 | 10.0 |  |
| Mg57Cu31.5Y8Nd3.5 | 426.0 | 501.0 | 778.0 | 12.0 |  |
| Mg57Cu31Y6.6Nd5.4 | 427.0 | 491.0 | 778.0 | 14.0 |  |
| Mg65.5Cu25.4Gd9 | 411.0 | 457.0 | 741.0 | 6.0 |  |
| Mg63.5Cu27.5Gd9 | 425.0 | 469.0 | 773.0 | 4.0 |  |
| Mg61.5Cu29.5Gd9 | 433.0 | 472.0 | 785.0 | 4.0 |  |
| Mg65Cu25Gd10 | 413.0 | 473.0 | 739.0 | 7.0 |  |
| Mg63Cu27Gd10 | 418.0 | 481.0 | 755.0 | 4.0 |  |
| Mg61Cu29Gd10 | 420.0 | 480.0 | 762.0 | 4.0 |  |
| Mg59Cu31Gd10 | 424.0 | 482.0 | 769.0 | 4.0 |  |
| Mg64.5Cu24.5Gd11 | 413.0 | 472.0 | 739.0 | 6.0 |  |
| Mg62.5Cu26.5Gd11 | 427.0 | 483.0 | 748.0 | 9.0 |  |
| Mg60.5Cu28.5Gd11 | 425.0 | 485.0 | 755.0 | 8.0 |  |
| Mg58.5Cu30.5Gd11 | 427.0 | 490.0 | 753.0 | 8.0 |  |
| Mg61Cu28Gd11 | 422.0 | 483.0 | 737.0 | 12.0 |  |
| La66Al14Cu20 | 395.0 | 449.0 | 731.0 | 2.0 |  |
| La55Al25Ni20 | 491.0 | 555.0 | 941.0 | 3.0 |  |
| La70Al14(Cu,Ni)16 | 404.0 | 429.0 | 763.0 | 0.5 |  |
| La68Al14(Cu,Ni)18 | 405.0 | 431.0 | 724.0 | 1.0 |  |
| La66Al14(Cu,Ni)20 | 405.0 | 431.0 | 674.0 | 1.5 |  |
| La62Al14(Cu,Ni)24 | 417.0 | 446.0 | 738.0 | 10.0 |  |
| La55Al25Ni10Cu10 | 467.4 | 547.2 | 835.0 | 5.0 |  |
| La55Al25Cu20 | 455.9 | 494.8 | 896.1 | 3.0 |  |
| La55Al25Ni5Cu10Co5 | 465.2 | 541.8 | 822.5 | 9.0 |  |
| La62Cu12Ni12Al14 | 423.0 | 452.0 | 744.0 | 12.0 |  |
| La62Al14Cu24 | 401.0 | 449.0 | 734.0 | 5.0 |  |
| La62Al14Cu22Ag2 | 401.0 | 455.0 | 722.0 | 5.0 |  |
| La62Al14Cu20Ag4 | 404.0 | 456.0 | 729.0 | 8.0 |  |
| La62Al14Cu19Ag5 | 405.0 | 456.0 | 730.0 | 5.0 |  |
| La62Al14Cu18Ag6 | 406.0 | 457.0 | 736.0 | 5.0 |  |
| La62Al14Cu17Ag7 | 406.0 | 458.0 | 739.0 | 5.0 |  |
| La62Al14Cu16Ag8 | 407.0 | 458.0 | 744.0 | 5.0 |  |
| La62Al14(Cu5/6Ag1/6)20(Ni1/2Co1/4 | 412.0 | 472.0 | 713.0 | 16.0 |  |
| La62Al14(Cu5/6Ag1/6)16(Ni1/2Co1/8 | 415.0 | 477.0 | 708.0 | 16.0 |  |
| La62Al14(Cu5/6Ag1/6)14(Ni1/2Co1/2)10 | 418.0 | 491.0 | 703.0 | 20.0 |  |
| La62Al14(Cu5/6Ag1/6)12(Ni1/2Co1/2)12 | 429.0 | 471.0 | 698.0 | 16.0 |  |
| La32Ce32Al16Ni5Cu15 | 403.0 | 451.0 | 712.0 | 10.0 |  |
| La32Ce32Al16Ni5Cu12Co3 | 406.0 | 455.0 | 709.0 | 10.0 |  |
| La32Ce32Al16Ni5Cu10Co5 | 413.0 | 467.0 | 718.0 | 12.0 |  |
| La32Ce32Al16Ni5Cu7Co8 | 416.0 | 471.0 | 739.0 | 10.0 |  |
| La32Ce32Al16Ni5Cu5Co10 | 424.0 | 472.0 | 767.0 | 10.0 |  |
| La62Al14(Cu0.5Ni0.5)24 | 423.0 | 452.0 | 744.0 | 12.0 |  |
| La55Al25Cu20 | 443.0 | 498.0 | 879.0 | 2.0 |  |
| La55Al20Ag5Cu20 | 429.0 | 503.0 | 823.0 | 4.0 |  |
| La55Al17.5Ag7.5Cu20 | 425.0 | 498.0 | 852.0 | 3.0 |  |
| La55Al15Ag10Cu20 | 416.0 | 483.0 | 787.0 | 2.0 |  |
| La55Al25Ag5Cu15 | 452.0 | 503.0 | 860.0 | 3.0 |  |
| La60Al15Ag5Cu20 | 401.0 | 481.0 | 759.0 | 5.0 |  |
| La62.5Al12.5Ag5Cu20 | 389.0 | 472.0 | 721.0 | 6.0 |  |
| La65Al10Ag5Cu20 | 380.0 | 458.0 | 716.0 | 5.0 |  |
| La62.5Al12.5Ag5Cu17.5Fe2.5 | 391.0 | 464.0 | 711.0 | 7.0 |  |
| La62.5Al12.5Ag5Cu15Fe5 | 390.0 | 445.0 | 713.0 | 6.0 |  |
| La62.5Al12.5Ag5Cu17.5Co2.5 | 393.0 | 473.0 | 712.0 | 8.0 |  |
| La62.5Al12.5Ag5Cu15Co5 | 397.0 | 474.0 | 700.0 | 9.0 |  |
| La70.0Al12.4(Cu,Ni)17.6 | 397.0 | 418.0 | 759.0 | 0.5 |  |
| La68.0Al13.2(Cu,Ni)18.8 | 400.0 | 426.0 | 743.0 | 1.0 |  |
| La66.0Al14.0(Cu,Ni)20.0 | 404.0 | 435.0 | 703.0 | 1.5 |  |
| La64.6Al14.6(Cu,Ni)20.8 | 406.0 | 442.0 | 706.0 | 5.0 |  |
| La63.1Al15.2(Cu,Ni)21.7 | 408.0 | 448.0 | 709.0 | 10.0 |  |
| La62.0Al15.6(Cu,Ni)22.4 | 410.0 | 453.0 | 712.0 | 11.0 |  |
| La61.4Al15.9(Cu,Ni)22.7 | 413.0 | 459.0 | 729.0 | 10.5 |  |
| La60.5Al16.3(Cu,Ni)23.2 | 414.0 | 465.0 | 734.0 | 8.0 |  |
| La59.6Al16.6(Cu,Ni)23.8 | 416.0 | 475.0 | 750.0 | 8.0 |  |
| La58.6Al17.0(Cu,Ni)24.4 | 421.0 | 489.0 | 774.0 | 5.0 |  |
| La57.6Al17.5(Cu,Ni)24.9 | 425.0 | 499.0 | 790.0 | 8.0 |  |
| La56.5Al17.9(Cu,Ni)25.6 | 433.0 | 492.0 | 823.0 | 2.0 |  |
| La55.4Al18.4(Cu,Ni)26.2 | 426.0 | 491.0 | 881.0 | 2.0 |  |
| Pd79.5Cu4Si16.5 | 635.0 | 675.0 | 1086.0 | 0.8 |  |
| Pd77.5Cu6Si16.5 | 637.0 | 678.0 | 1058.0 | 1.5 |  |
| Pd81.5Cu2Si6.5 | 633.0 | 670.0 | 1097.0 | 2.0 |  |
| Pd77Cu6Si17 | 642.0 | 686.0 | 1128.0 | 2.0 |  |
| Pd73.5Cu10Si16.5 | 645.0 | 685.0 | 1136.0 | 2.0 |  |
| Pd71.5Cu12Si16.5 | 652.0 | 680.0 | 1154.0 | 2.0 |  |
| Pd40Ni40P20 | 590.0 | 671.0 | 991.0 | 25.0 |  |
| Pd40Cu30Ni10P20 | 576.9 | 655.8 | 836.0 | 72.0 |  |
| Pd77Cu6Si17 | 642.4 | 686.4 | 1128.0 | 2.0 |  |
| Pd79Cu6Si10P5 | 609.0 | 682.0 | 995.0 | 5.0 |  |
| Pd79Cu5Ag1Si10P5 | 614.0 | 684.0 | 1001.0 | 4.0 |  |
| Pd79Cu4Ag2Si10P5 | 613.0 | 684.0 | 1005.0 | 5.0 |  |
| Pd79Cu3Ag3Si10P5 | 610.0 | 683.0 | 1005.0 | 5.0 |  |
| Pd79Cu2Ag4Si10P5 | 611.0 | 676.0 | 1006.0 | 7.0 |  |
| Ti50Ni30Cu32Sn3 | 686.0 | 759.0 | 1283.0 | 1.0 |  |
| Ti50Ni15Cu25Sn3Be7 | 688.0 | 733.0 | 1207.0 | 2.0 |  |
| Ti45Ni15Cu25Sn3Be7Zr5 | 680.0 | 741.0 | 1142.0 | 5.0 |  |
| Ti40Zr25Ni8Cu9Be18 | 621.0 | 668.0 | 1009.0 | 8.0 |  |
| Ti50Cu42.5Ni7.5 | 670.0 | 708.0 | 1226.0 | 0.2 |  |
| Ti47.5Zr2.5Cu42.5Ni7.5 | 673.0 | 720.0 | 1225.0 | 1.5 |  |
| Ti42.5Zr2.5Hf5Cu42.5Ni7.5 | 677.0 | 726.0 | 1203.0 | 2.5 |  |
| Ti41.5Zr2.5Hf5Cu42.5Ni7.5Si1 | 680.0 | 730.0 | 1199.0 | 5.0 |  |
| Ti55Zr10Cu9Ni8Be18 | 629.0 | 667.0 | 1013.0 | 6.0 |  |
| Ti50Zr15Cu9Ni8Be18 | 622.0 | 662.0 | 1009.0 | 6.0 |  |
| Ti50Ni24Cu20B1Si2Sn3 | 726.0 | 800.0 | 1310.0 | 1.0 |  |
| Ti34Zr11Cu47Ni8 | 698.4 | 727.2 | 1169.0 | 4.5 |  |
| Ti41.5Zr2.5Hf5Cu42.5Ni7.5Si1 | 684.6 | 719.9 | 1206.0 | 2.0 |  |
| Ti41.5Zr2.5Hf5Cu37.5Ni7.5Si1Sn5 | 693.3 | 757.5 | 1176.0 | 6.0 |  |
| Ti40Zr10Cu40Pd10 | 660.0 | 709.0 | 1184.0 | 4.0 |  |
| Ti40Zr10Cu34Pd16 | 672.0 | 723.0 | 1231.0 | 4.0 |  |
| Ti40Zr10Cu32Pd18 | 683.0 | 740.0 | 1272.0 | 3.0 |  |
| Ti40Zr10Cu30Pd20 | 687.0 | 747.0 | 1279.0 | 3.0 |  |
| Ti53Cu15Ni18.5Al7Si3Sc3B0.5 | 709.0 | 767.0 | 1240.0 | 2.0 |  |
| Ti53Cu15Ni18.5Al7Si3Hf3B0.5 | 695.0 | 749.0 | 1230.0 | 2.0 |  |
| Ti40Zr10Cu38Pd12 | 666.0 | 715.0 | 1189.0 | 6.0 |  |
| Ti40Zr10Cu36Pd14 | 669.0 | 718.0 | 1191.0 | 6.0 |  |
| Pr68Cu25Al7 | 382.0 | 402.0 | 705.0 | 1.5 |  |
| Pr68(Cu,Ni)25Al7 | 399.0 | 416.0 | 703.0 | 1.5 |  |
| Pr72(Cu,Ni)25Al3 | 367.0 | 402.0 | 743.0 | 1.5 |  |
| Pr72(Cu,Ni)21Al7 | 395.0 | 410.0 | 760.0 | 1.5 |  |
| Y56Al24Co20 | 636.0 | 690.0 | 1078.0 | 1.5 |  |
| Y36Sc20Al24Co20 | 645.0 | 760.0 | 1034.0 | 25.0 |  |
| Y36Sc20Al24Co10Ni10 | 645.0 | 731.0 | 1010.0 | 25.0 |  |
| Co50Cr15Mo14C15B6 | 819.0 | 895.0 | 1417.0 | 2.0 |  |
| Co48Cr15Mo14C15B6Er2 | 848.0 | 933.0 | 1394.0 | 10.0 |  |
| Co43Fe20Ta5.5B31.5 | 910.0 | 982.0 | 1526.0 | 2.0 |  |
| [(Co0.9Fe0.1)0.75B0.2Si0.05]96Nb4 | 803.0 | 843.0 | 1457.0 | 2.0 |  |
| [(Co0.8Fe0.2)0.75B0.2Si0.05]96Nb4 | 813.0 | 853.0 | 1445.0 | 2.5 |  |
| [(Co0.7Fe0.3)0.75B0.2Si0.05]96Nb4 | 820.0 | 860.0 | 1430.0 | 3.5 |  |
| [(Co0.6Fe0.4)0.75B0.2Si0.05]96Nb4 | 823.0 | 865.0 | 1418.0 | 4.0 |  |
| Au55Cu25Si20 | 348.0 | 383.0 | 654.0 | 0.5 |  |
| Au46Ag5Cu29Si20 | 395.0 | 420.0 | 664.0 | 1.0 |  |
| Au52Pd2.3Cu29.2Si16.5 | 393.0 | 427.0 | 651.0 | 2.0 |  |
| Au49Ag5.5Pd2.3Cu26.9Si16.3 | 401.0 | 459.0 | 644.0 | 5.0 |  |
| Hf47Cu29.25Ni9.75Al14 | 790.0 | 875.0 | 1278.0 | 10.0 |  |
| Hf48Cu29.25Ni9.75Al13 | 785.0 | 874.0 | 1280.0 | 10.0 |  |
| Hf51Cu27.75Ni9.25Al12 | 777.0 | 872.0 | 1344.0 | 8.0 |  |
| Gd60Co25Al15 | 572.0 | 617.0 | 952.0 | 5.0 |  |
| Gd60Ni15Al25 | 603.0 | 648.0 | 1006.0 | 4.0 |  |
| Zr65Al7.5Cu17.5Ni10 | 656.5 | 735.6 | 1167.0 | 16.0 |  |
| Zr57Ti5Al10Cu20Ni8 | 676.7 | 720.0 | 1145.0 | 10.0 |  |
| Zr65.5Al5.6Ni6.5Cu22.4 | 630.0 | 733.0 | 1211.0 | 3.0 |  |
| Zr41.2Ti13.8Cu12.5Ni10Be22.5 | 623.0 | 672.0 | 996.0 | 50.0 |  |
| Zr54Cu46 | 696.0 | 746.0 | 1201.0 | 2.0 |  |
| Zr47Cu46Al7 | 705.0 | 781.0 | 1163.0 | 3.0 |  |
| Zr41Ti14Cu12.5Ni8Be22.5C2 | 628.0 | 683.0 | 997.0 | 5.0 |  |
| Zr41Ti14Cu12.5Ni2Be22.5C8[ | 629.0 | 727.0 | 992.0 | 3.0 |  |
| Zr26Ti10Cu8Ni8Be20Y4Mg24 | 650.0 | 700.0 | 951.0 | 5.0 |  |
| Zr40Ti15Cu11Ni11Be21.5Y1Mg0.5 | 630.0 | 674.0 | 975.0 | 5.0 |  |
| Zr48Nb8Cu14Ni12Be18 | 656.0 | 724.0 | 1072.0 | 8.0 |  |
| Zr48Nb8Cu12Fe8Be24 | 658.0 | 751.0 | 1071.0 | 8.0 |  |
| Zr36Nb12Cu10Ni8Be20Y2Mg12 | 653.0 | 733.0 | 1029.0 | 5.0 |  |
| Zr36Nb12Cu10Ni6Fe2Be20Y2Mg12 | 670.0 | 712.0 | 1029.0 | 5.0 |  |
| Zr54Al15Ni10Cu19Y2 | 714.0 | 787.0 | 1112.0 | 5.0 |  |
| Zr53Al14Ni10Cu19Y4 | 668.0 | 766.0 | 1069.0 | 5.0 |  |
| Zr51Cu20.7Ni12Al16.3 | 722.0 | 800.0 | 1132.0 | 3.0 |  |
| Zr55Al20Co25 | 761.0 | 840.0 | 1245.0 | 2.5 |  |
| Zr48Cu45Al7 | 698.0 | 758.0 | 1208.0 | 5.0 |  |
| Zr48Cu43Al7Ag2 | 700.0 | 761.0 | 1152.0 | 12.0 |  |
| Zr48Cu42Al7Ag3 | 700.0 | 763.0 | 1135.0 | 10.0 |  |
| Zr48Cu40Al7Ag5 | 699.0 | 769.0 | 1121.0 | 10.0 |  |
| Zr48Cu37Al7Ag8 | 698.0 | 765.0 | 1125.0 | 10.0 |  |
| Zr48Cu36Ag8Al8 | 690.0 | 791.0 | 1143.0 | 25.0 |  |
| Zr48Cu34Pd2Ag8Al8 | 699.0 | 794.0 | 1140.0 | 30.0 |  |
| Zr50Cu48Ag2 | 668.0 | 719.0 | 1192.0 | 2.0 |  |
| Zr50Cu45Ag5 | 669.0 | 728.0 | 1188.0 | 4.0 |  |
| Zr50Cu43Ag7 | 669.0 | 727.0 | 1171.0 | 4.0 |  |
| Zr50Cu40Ag10 | 667.0 | 733.0 | 1177.0 | 5.0 |  |
| Zr50Cu38Ag12 | 663.0 | 734.0 | 1187.0 | 4.0 |  |
| (Fe0.75B0.2Si0.05)96Nb4 | 835.0 | 880.0 | 1475.0 | 1.5 |  |
| [(Fe0.9Co0.1)0.75B0.2Si0.05]96Nb4 | 832.0 | 877.0 | 1460.0 | 2.0 |  |
| [(Fe0.8Co0.2)0.75B0.2Si0.05]96Nb4 | 830.0 | 880.0 | 1431.0 | 2.5 |  |
| [(Fe0.7Co0.3)0.75B0.2Si0.05]96Nb4 | 828.0 | 878.0 | 1413.0 | 3.5 |  |
| [(Fe0.6Co0.4)0.75B0.2Si0.05]96Nb4 | 825.0 | 875.0 | 1407.0 | 4.0 |  |
| [(Fe0.5Co0.5)0.75B0.2Si0.05]96Nb4 | 820.0 | 870.0 | 1397.0 | 5.0 |  |
| Fe48Cr15Mo14C15B6Y2 | 839.0 | 886.0 | 1464.0 | 7.0 |  |
| Fe45Co3Cr15Mo14C15B6Y2 | 834.0 | 880.0 | 1446.0 | 8.0 |  |
| Fe43Co5Cr15Mo14C15B6Y2 | 835.0 | 872.0 | 1442.0 | 9.0 |  |
| Fe39Co9Cr15Mo14C15B6Y2 | 838.0 | 888.0 | 1466.0 | 10.0 |  |
| Fe41Co7Cr15Mo14C15B6Y2 | 838.0 | 875.0 | 1436.0 | 16.0 |  |
| Fe61B15Mo7Zr8Co7Y2 | 904.6 | 916.4 | 1490.0 | 5.0 |  |
| Fe61B15Mo7Zr8Co6Y2Al1 | 899.5 | 955.6 | 1495.0 | 5.0 |  |
| Fe61B15Mo7Zr8Co5Y2Cr2 | 901.1 | 958.9 | 1490.0 | 5.0 |  |
| Fe56Mn5Cr7Mo12Er2C12B6 | 793.0 | 832.0 | 1401.0 | 8.0 |  |
| Fe63C15Mo14Er2B6 | 771.0 | 830.0 | 1389.0 | 3.0 |  |
| Fe58Cr5Mo14Er2C15B6 | 793.0 | 829.0 | 1416.0 | 6.0 |  |
| Fe48Cr15Mo14Er2C15B6 | 844.0 | 880.0 | 1446.0 | 8.0 |  |
| Fe68.3C6.9Si2.5B6.7P8.8Cr2.2Mo2.5Al2.1 | 795.0 | 835.0 | 1316.0 | 4.0 |  |
| Fe72Nb4B20Si4 | 842.0 | 880.0 | 1420.0 | 2.0 |  |
| (Fe72Nb4B20Si4)99Y1 | 855.0 | 881.0 | 1419.0 | 2.0 |  |
| (Fe72Nb4B20Si4)98Y2 | 855.0 | 903.0 | 1416.0 | 2.0 |  |
| (Fe72Nb4B20Si4)97Y3 | 859.0 | 915.0 | 1416.0 | 4.0 |  |
| (Fe72Nb4B20Si4)96Y4 | 905.0 | 933.0 | 1424.0 | 3.0 |  |
| Fe74Nb6Y3B17 | 831.0 | 879.0 | 1391.0 | 2.0 |  |
| (((Fe0.6Co0.4)0.75B0.2Si0.05)0.96Nb0.04)100 | 826.0 | 870.0 | 1452.0 | 4.0 |  |
| (((Fe0.6Co0.4)0.75B0.2Si0.05)0.96Nb0.04)99Cr1 | 827.0 | 871.0 | 1462.0 | 4.0 |  |
| (((Fe0.6Co0.4)0.75B0.2Si0.05)0.96Nb0.04)98Cr2 | 830.0 | 873.0 | 1469.0 | 4.0 |  |
| (((Fe0.6Co0.4)0.75B0.2Si0.05)0.96Nb0.04)97Cr3 | 831.0 | 874.0 | 1474.0 | 3.5 |  |
| (((Fe0.6Co0.4)0.75B0.2Si0.05)0.96Nb0.04)96Cr4 | 833.0 | 874.0 | 1481.0 | 3.0 |  |
| Fe76Si9B10P5 | 780.0 | 832.0 | 1258.0 | 2.5 |  |
| Fe27Co40Zr3Ti3Mo1.5Si1.5B24 | 811.0 | 856.0 | 1379.0 | 1.5 |  |
| (Fe81.5Si3.8C14Tm0.7)92.37P7.63 | 687.0 | 752.0 | 1284.0 | 1.0 |  |
| (Fe81.5Si3.8C14Tm0.7)90.9P9.1 | 717.0 | 767.0 | 1318.0 | 1.0 |  |
| Fe65.5Cr4Mo4Ga4P12C5B5.5 | 745.0 | 806.0 | 1322.0 | 3.0 |  |
| Fe76Mo4(P0.45,C0.2,B0.2,Si0.15)20 | 744.0 | 788.0 | 1245.0 | 4.0 |  |
| Fe66Co10Mo4(P0.45,C0.2,B0.2,Si0.15)20 | 744.0 | 788.0 | 1221.0 | 6.0 |  |
| Fe56Co20Mo4(P0.45,C0.2,B0.2,Si0.15)20 | 736.0 | 778.0 | 1220.0 | 5.0 |  |
| Fe46Co30Mo4(P0.45,C0.2,B0.2,Si0.15)20 | 734.0 | 775.0 | 1233.0 | 3.0 |  |
| Fe72Y6B22 | 898.0 | 944.0 | 1419.0 | 2.0 |  |
| Fe71Ni1Y6B22 | 883.0 | 926.0 | 1507.0 | 2.0 |  |
| Fe70Ni2Y6B22 | 880.0 | 925.0 | 1509.0 | 2.0 |  |
| Fe69Ni3Y6B22 | 874.0 | 910.0 | 1503.0 | 1.5 |  |
| Fe68Ni4Y6B22 | 872.0 | 907.0 | 1470.0 | 1.5 |  |
| Fe67Ni5Y6B22 | 866.0 | 891.0 | 1469.0 | 1.0 |  |
| Fe70Co2Y6B22 | 898.0 | 944.0 | 1420.0 | 2.0 |  |
| Fe68Co4Y6B22 | 896.0 | 941.0 | 1414.0 | 2.0 |  |
| Fe66Co6Y6B22 | 887.0 | 925.0 | 1509.0 | 2.0 |  |
| Fe64Co8Y6B22 | 884.0 | 927.0 | 1505.0 | 2.5 |  |
| Fe62Co10Y6B22 | 885.0 | 932.0 | 1503.0 | 2.5 |  |
| Fe60Co12Y6B22 | 881.0 | 924.0 | 1498.0 | 2.5 |  |
| Fe58Co14Y6B22 | 880.0 | 925.0 | 1485.0 | 2.5 |  |
| Fe56Co16Y6B22 | 882.0 | 927.0 | 1494.0 | 2.5 |  |
| Fe71Mo1Y6B22 | 902.0 | 960.0 | 1517.0 | 2.5 |  |
| Fe70Mo2Y6B22 | 907.0 | 969.0 | 1508.0 | 3.5 |  |
| Fe69Mo3Y6B22 | 908.0 | 958.0 | 1488.0 | 6.0 |  |
| Fe68Mo4Y6B22 | 915.0 | 944.0 | 1488.0 | 6.5 |  |
| Fe67Mo5Y6B22 | 920.0 | 941.0 | 1483.0 | 3.5 |  |
| Fe76Mo2Ga2P10C4B4Si2 | 736.0 | 788.0 | 1247.0 | 2.0 |  |
| Fe74Mo4Ga2P10C4B4Si2 | 740.0 | 790.0 | 1276.0 | 1.5 |  |
| Fe75Mo2Ga3P10C4B4Si2 | 738.0 | 798.0 | 1230.0 | 2.5 |  |
| Fe73Mo4Ga3P10C4B4Si2 | 744.0 | 801.0 | 1283.0 | 2.0 |  |
| Fe79P10C4B4Si3 | 740.0 | 774.0 | 1263.0 | 1.0 |  |
| Fe78Mo1P10C4B4Si3 | 742.0 | 780.0 | 1268.0 | 1.5 |  |
| Fe77Mo2P10C4B4Si3 | 742.0 | 783.0 | 1264.0 | 2.5 |  |
| Fe76Mo3P10C4B4Si3 | 750.0 | 793.0 | 1250.0 | 3.5 |  |
| Fe75Mo4P10C4B4Si3 | 752.0 | 799.0 | 1227.0 | 4.0 |  |
| Fe74Mo5P10C4B4Si3 | 758.0 | 799.0 | 1263.0 | 3.0 |  |
| (Fe0.75B0.15Si0.10)99Zr1 | 867.0 | 919.0 | 1469.0 | 0.8 |  |
| (Fe0.75B0.15Si0.10)99Nb1 | 815.0 | 858.0 | 1455.0 | 0.5 |  |
| (Fe0.75B0.15Si0.10)98Nb2 | 812.0 | 870.0 | 1425.0 | 1.0 |  |
| (Fe0.75B0.15Si0.10)96Nb4 | 835.0 | 885.0 | 1369.0 | 1.5 |  |
| Ni60Nb30Ta10 | 934.0 | 961.0 | 1559.0 | 2.0 |  |
| Ni61Zr28Nb7Al4 | 848.0 | 898.0 | 1348.0 | 1.0 |  |
| Ni61Zr22Nb7Al4Ta6 | 867.0 | 927.0 | 1379.0 | 2.0 |  |
| Ni59Zr20Ti16Si5 | 830.0 | 876.0 | 1304.0 | 2.0 |  |
| Ni59Zr20Ti16Sn5 | 819.0 | 854.0 | 1288.0 | 1.0 |  |
| Ni59Zr20Ti16Si2Sn3 | 821.0 | 877.0 | 1272.0 | 3.0 |  |
| Ni42Ti20Zr25Al8Cu5 | 748.0 | 803.0 | 1366.0 | 0.5 |  |
| Ni42Ti20Zr22.5Al8Cu5Si2.5 | 767.0 | 833.0 | 1367.0 | 2.0 |  |
| Ni42Ti20Zr21.5Al8Cu5Si3.5 | 774.0 | 846.0 | 1366.0 | 2.5 |  |
| Ni42Ti20Zr20.5Al8Cu5Si4.5 | 763.0 | 856.0 | 1364.0 | 2.0 |  |
| Ni42Ti19Zr22.5Al8Cu5Si3.5 | 780.0 | 846.0 | 1363.0 | 3.0 |  |
| Ni60Nb40 | 891.0 | 924.0 | 1478.0 | 1.0 |  |
| Ni60Nb35Zr5 | 887.0 | 911.0 | 1458.0 | 1.5 |  |
| Ni60Nb30Zr10 | 875.0 | 902.0 | 1413.0 | 2.0 |  |
| Ni60Nb25Zr15 | 860.0 | 891.0 | 1390.0 | 1.5 |  |
| Ni60Nb20Zr20 | 853.0 | 891.0 | 1391.0 | 0.5 |  |
| Ni60Zr20Ti2.5Nb12.5Al5 | 836.0 | 897.0 | 1378.0 | 2.0 |  |
| Ni60Zr20Ti5Nb10Al5 | 826.0 | 896.0 | 1379.0 | 2.0 |  |
| Ni60Zr20Ti7.5Nb7.5Al5 | 824.0 | 885.0 | 1385.0 | 2.0 |  |
| (Ni0.75B0.2Si0.05)96Nb4 | 770.0 | 795.0 | 1446.0 | 0.5 |  |
| ((Ni0.9Fe0.1)0.75B0.2Si0.05)96Nb4 | 762.0 | 795.0 | 1408.0 | 1.0 |  |
| ((Ni0.8Fe0.2)0.75B0.2Si0.05)96Nb4 | 755.0 | 795.0 | 1381.0 | 2.0 |  |
| ((Ni0.7Fe0.3)0.75B0.2Si0.05)96Nb4 | 750.0 | 795.0 | 1356.0 | 2.5 |  |
| ((Ni0.6Fe0.4)0.75B0.2Si0.05)96Nb4 | 745.0 | 795.0 | 1348.0 | 3.0 |  |
| Ce70Al10Cu20 | 341.0 | 408.0 | 722.0 | 2.0 |  |
| Ce70Al15Cu15 | 364.0 | 406.0 | 686.0 | 2.0 | ^10^ |
| Ce65Al15Cu20 | 363.0 | 425.0 | 773.0 | 2.0 |  |
| Ce60Al20Cu20 | 396.0 | 444.0 | 830.0 | 3.0 |  |
| Ce55Al25Cu20 | 439.0 | 479.0 | 825.0 | 1.0 |  |
| Ce60Al20Co20 | 424.0 | 468.0 | 798.0 | 1.0 |  |
| Ce70Al10Ni20 | 373.0 | 399.0 | 775.0 | 1.0 |  |
| Ce70Al48Ni15 | 368.0 | 387.0 | 738.0 | 1.0 |  |
| Ce68Al10Cu20Fe2 | 352.0 | 423.0 | 708.0 | 5.0 |  |
| Ce69.8Al10Cu20Co0.2 | 339.0 | 414.0 | 721.0 | 8.0 |  |
| Ce69.5Al10Cu20Co0.5 | 337.0 | 419.0 | 716.0 | 10.0 |  |
| Ce69Al10Cu20Co1 | 340.0 | 421.0 | 713.0 | 10.0 |  |
| Ce68Al10Cu20Co2 | 352.0 | 419.0 | 716.0 | 10.0 |  |
| Ce65Al10Cu20Co5 | 363.0 | 414.0 | 695.0 | 8.0 |  |
| Ce68Al10Cu20Ni2 | 352.0 | 421.0 | 710.0 | 5.0 |  |
| Ce60Al10Cu20Ni10 | 374.0 | 441.0 | 672.0 | 1.0 |  |
| Ce69Al10Cu20Nb1 | 352.0 | 412.0 | 728.0 | 10.0 |  |
| Ce68Al10Cu20Nb2 | 345.0 | 421.0 | 721.0 | 8.0 |  |
| Ce67Al10Cu20Nb3 | 355.0 | 404.0 | 723.0 | 5.0 |  |
| Ce70Al10Cu19Zn1 | 343.0 | 391.0 | 743.0 | 1.0 |  |
| Ce70Al10Cu18Zn2 | 345.0 | 399.0 | 730.0 | 2.0 |  |
| Ce70Al10Cu17Zn3 | 341.0 | 412.0 | 733.0 | 3.0 |  |
| Ce70Al10Cu18Bi2 | 348.0 | 408.0 | 708.0 | 1.0 |  |
| Ce68Al10Cu20Si2 | 352.0 | 413.0 | 721.0 | 3.0 |  |
| Ce68Al10Cu20C2 | 352.0 | 406.0 | 723.0 | 2.0 |  |
| Ce68Al10Cu20B2 | 346.0 | 393.0 | 731.0 | 2.0 |  |
| Zr62Cu23Fe5Al10 | 651.0 | 751.0 | 1186.0 | 3.0 |  |
| (Zr0.62Cu0.23Fe0.05Al0.10)99Ag1 | 656.0 | 753.0 | 1175.0 | 4.0 | ^11^ |
| (Zr0.62Cu0.23Fe0.05Al0.10)97Ag3 | 658.0 | 743.0 | 1181.0 | 10.0 |  |
| (Zr0.62Cu0.23Fe0.05Al0.10)95Ag5 | 663.0 | 747.0 | 1189.0 | 5.0 |  |
| (Zr0.62Cu0.23Fe0.05Al0.10)93Ag7 | 664.0 | 751.0 | 1191.0 | 4.0 |  |
| Zr56Co28Al16 | 742.0 | 791.0 | 1234.0 | 6.0 | ^12^ |
| Zr54Co28Al16Y2 | 731.0 | 781.0 | 1238.0 | 5.0 |  |
| Zr51Co28Al16Y5 | 728.0 | 772.0 | 1212.0 | 5.0 |  |
| Zr44Co28Al16Y12 | 718.0 | 758.0 | 1233.0 | 6.0 |  |
| Zr70Al8Cu16Ni6 | 641.0 | 707.0 | 1179.0 | 6.0 | ^13^ |
| Zr70Al8Cu13.5Ni8.5 | 625.0 | 707.0 | 1171.0 | 10.0 |  |
| Zr70Al8Cu11Ni11 | 633.0 | 706.0 | 1175.0 | 8.0 |  |
| Zr70Al8Cu6Ni16 | 631.0 | 705.0 | 1205.0 | 8.0 |  |
| Zr55Al20Co20Cu5 | 737.0 | 815.0 | 1285.0 | 5.0 | ^14^ |
| (Zr0.55Al0.20Co0.20Cu0.05)99Ag1 | 739.0 | 813.0 | 1275.0 | 10.0 |  |
| (Zr0.55Al0.20Co0.20Cu0.05)97Ag3 | 740.0 | 805.0 | 1246.0 | 16.0 |  |
| (Zr0.55Al0.20Co0.20Cu0.05)95Ag5 | 747.0 | 806.0 | 1220.0 | 16.0 |  |
| (Zr0.55Al0.20Co0.20Cu0.05)93Ag7 | 753.0 | 806.0 | 1222.0 | 14.0 |  |
| Zr60Ni25Al15 | 694.0 | 787.0 | 1291.0 | 15.0 | ^15^ |
| Zr62Ni25Al13 | 682.0 | 773.0 | 1279.0 | 12.0 |  |
| Zr58Ni25Al15Nb2 | 712.0 | 801.0 | 1264.0 | 16.0 |  |
| Zr56Ni25Al15Nb4 | 717.0 | 801.0 | 1271.0 | 20.0 |  |
| Zr58Ni25Al13Nb4 | 705.0 | 771.0 | 1202.0 | 15.0 |  |
| Zr53Al16Co31 | 752.0 | 812.0 | 1303.0 | 3.0 | ^16^ |
| Zr53Al16(Co0.8Ag0.2)31 | 754.0 | 792.0 | 1234.0 | 16.0 |  |
| Zr53Al16(Co0.75Ag0.25)31 | 755.0 | 791.0 | 1234.0 | 20.0 |  |
| Zr53Al16(Co0.7Ag0.3)31 | 756.0 | 791.0 | 1234.0 | 16.0 |  |
| Zr53Al16(Co0.6Ag0.4)31 | 759.0 | 790.0 | 1231.0 | 14.0 |  |
| Zr53Co23.5Al23.5 | 772.0 | 841.0 | 1296.0 | 3.0 | ^17^ |
| Zr53Co22.5Al23.5Ag1 | 771.0 | 840.0 | 1309.0 | 3.0 |  |
| Zr53Co20.5Al23.5Ag3 | 773.0 | 839.0 | 1297.0 | 5.0 |  |
| Zr53Co18.5Al23.5Ag5 | 776.0 | 839.0 | 1245.0 | 10.0 |  |
| Zr53Co16.5Al23.5Ag7 | 770.0 | 830.0 | 1261.0 | 5.0 |  |
| Zr53Co14.5Al23.5Ag9 | 769.0 | 821.0 | 1265.0 | 5.0 |  |
| Zr60Nb5Cu22.5Pd5Al7.5 | 683.0 | 721.0 | 1226.0 | 4.0 | ^18^ |
| Zr60Ti6Cu19Fe5Al10 | 658.0 | 724.0 | 1155.0 | 5.0 |  |
| Zr60Nb5Cu20Fe5Al10 | 623.0 | 722.0 | 1098.0 | 5.0 |  |
| Ag30.8Mg23.1Ca30.8Cu15.4 | 413.0 | 432.0 | 803.0 | 2.5 | ^19^ |
| Ag30.8Mg30.8Ca30.8Cu7.7 | 407.0 | 427.0 | 809.0 | 2.0 |  |
| Ag38.5Mg23Ca30.8Cu7.7 | 384.0 | 416.0 | 854.0 | 2.0 |  |
| Ag38.5Mg30.8Ca23.1Cu7.7 | 387.0 | 420.0 | 833.0 | 3.0 |  |
| Ag46.2Mg23.2Ca23Cu7.7 | 398.0 | 430.0 | 825.0 | 2.0 |  |
| Ag50Mg11.5Ca30.8Cu7.7 | 452.0 | 487.0 | 809.0 | 1.0 |  |
| Ag50Mg19.2Ca23.1Cu7.7 | 426.0 | 466.0 | 797.0 | 1.2 |  |
| Ca30Mg25Cu45 | 387.0 | 430.0 | 798.0 | 2.5 |  |
| Ca35Mg25Cu40 | 386.0 | 429.0 | 748.0 | 3.5 |  |
| Ca33.3Mg33.3Ca33.3 | 391.0 | 406.0 | 731.0 | 3.0 |  |
| Cu36.4Mg27.2Ca36.4 | 383.0 | 412.0 | 706.0 | 8.0 |  |
| Cu36.4Mg31.8Ca31.8 | 388.0 | 400.0 | 742.0 | 8.0 |  |
| Cu36.4Mg36.4Ca27.2 | 382.0 | 400.0 | 750.0 | 5.0 |  |
| Cu40.9Mg18.2Ca40.9 | 393.0 | 431.0 | 717.0 | 3.0 |  |
| Cu40.9Mg22.7Ca36.4 | 396.0 | 432.0 | 719.0 | 5.0 |  |
| Cu40.9Mg27.3Ca31.8 | 391.0 | 430.0 | 745.0 | 4.0 |  |
| Cu40.9Mg31.8Ca27.3 | 392.0 | 414.0 | 785.0 | 3.0 |  |
| Cu40.9Mg36.4Ca22.7 | 394.0 | 414.0 | 821.0 | 1.5 |  |
| Cu45.5Mg18.1Ca36.4 | 397.0 | 426.0 | 735.0 | 3.0 |  |
| Cu45.5Mg22.7Ca31.8 | 388.0 | 427.0 | 793.0 | 3.0 |  |
| Cu45.5Mg27.3Ca27.2 | 395.0 | 410.0 | 831.0 | 1.5 |  |
| Cu45.5Mg31.8Ca22.7 | 389.0 | 410.0 | 843.0 | 1.25 |  |
| Cu45.5Mg36.4Ca18.1 | 391.0 | 420.0 | 851.0 | 2.0 |  |
| Cu50Mg18.2Ca31.8 | 392.0 | 421.0 | 828.0 | 2.0 |  |
| Cu50Mg22.7Ca27.3 | 392.0 | 410.0 | 864.0 | 1.5 |  |
| Cu54.5Mg18.2Ca27.3 | 401.0 | 423.0 | 884.0 | 1.0 |  |
| (Cu50Zr43Al7)98.5Si1.5 | 737.0 | 798.0 | 1243.0 | 4.0 |  |
| (Cu50Zr43Al7)98Si2 | 740.0 | 800.0 | 1256.0 | 3.0 |  |
| (Cu50Zr43Al7)99.5Si0.5 | 719.0 | 791.0 | 1214.0 | 10.0 |  |
| (Cu50Zr43Al7)99Si1 | 720.0 | 797.0 | 1226.0 | 12.0 |  |
| Fe36Co36B19.2Si4.8Nb4 | 817.0 | 856.0 | 1359.0 | 2.0 |  |
| Fe55.8Co14.2Nb6B24 | 821.0 | 868.0 | 1477.0 | 2.0 |  |
| Fe56.05Co13.45Nb5.5B25 | 821.0 | 879.0 | 1510.0 | 2.5 |  |
| Fe56.8Co14.2Nb5B24 | 823.0 | 868.0 | 1496.0 | 2.0 |  |
| Fe66W6Y6B22 | 897.0 | 981.0 | 1497.0 | 2.0 |  |
| Fe67W5Y6B22 | 882.0 | 970.0 | 1497.0 | 3.0 |  |
| Fe68W4Y6B22 | 874.0 | 971.0 | 1504.0 | 4.0 |  |
| Fe69W3Y6B22 | 868.0 | 973.0 | 1505.0 | 4.0 |  |
| Fe70Nb4Hf3Y3B20 | 850.0 | 924.0 | 1437.0 | 3.5 |  |
| Fe70W2Y6B22 | 867.0 | 970.0 | 1521.0 | 3.0 |  |
| Fe71Mo3Nb2P12C10B2 | 724.0 | 766.0 | 1305.0 | 1.0 |  |
| Fe71Nb4Hf3Y2B20 | 850.0 | 928.0 | 1432.0 | 4.0 |  |
| Fe71W1Y6B22 | 845.0 | 956.0 | 1423.0 | 3.0 |  |
| Fe71Mo2Nb3P12C10B2 | 719.0 | 757.0 | 1305.0 | 1.0 |  |
| Fe71Mo4Nb1P12C10B2 | 729.0 | 777.0 | 1296.0 | 2.0 |  |
| Fe71Nb6B23 | 819.0 | 865.0 | 1494.0 | 1.5 |  |
| Fe72C7Si3.3B5P8.7Ga4 | 782.0 | 801.0 | 1290.0 | 2.0 |  |
| Fe72Nb4Hf3Y1B20 | 841.0 | 916.0 | 1446.0 | 3.0 |  |
| Fe73Nb4Hf3B20 | 836.0 | 899.0 | 1448.0 | 2.0 |  |
| Fe74C7Si3.3B5P8.7Ga2 | 784.0 | 800.0 | 1283.0 | 3.0 |  |
| Fe75C7Si3.3B5P8.7Ga1 | 781.0 | 794.0 | 1282.0 | 3.0 |  |
| Fe76C7Si3.3B5P8.7 | 779.0 | 795.0 | 1292.0 | 1.0 |  |
| (Fe0.72Tb0.03B0.2Si0.05)96Nb4 | 860.0 | 940.0 | 1422.0 | 3.0 |  |
| (Fe0.73Tb0.02B0.2Si0.05)96Nb4 | 844.0 | 914.0 | 1440.0 | 2.5 |  |
| (Fe0.74Tb0.01B0.2Si0.05)96Nb4 | 836.0 | 882.0 | 1459.0 | 1.0 |  |
| (Fe0.68Tb0.07B0.2Si0.05)96Nb4 | 959.0 | 1019.0 | 1438.0 | 2.0 |  |
| (Fe0.69Tb0.06B0.2Si0.05)96Nb4 | 952.0 | 1012.0 | 1411.0 | 3.0 |  |
| (Fe0.7Tb0.05B0.2Si0.05)96Nb4 | 935.0 | 995.0 | 1396.0 | 3.5 |  |
| (Fe0.71Tb0.04B0.2Si0.05)96Nb4 | 869.0 | 969.0 | 1397.0 | 3.5 |  |
| (Fe72Mo4B24)93Dy7 | 880.0 | 957.0 | 1406.0 | 2.0 |  |
| (Fe72Mo4B24)94Dy6 | 854.0 | 945.0 | 1400.0 | 3.0 |  |
| (Fe72Mo4B24)95Dy5 | 853.0 | 942.0 | 1401.0 | 2.0 |  |
| (Fe72Mo4B24)96Dy4 | 848.0 | 923.0 | 1396.0 | 2.0 |  |
| (Fe70Co20)71Nb6B23 | 809.0 | 864.0 | 1475.0 | 2.0 |  |
| [(Fe0.5Co0.5)72Mo4B24]94Dy6 | 831.0 | 912.0 | 1365.0 | 2.0 |  |
| [(Fe0.6Co0.4)72Mo4B24]94Dy6 | 847.0 | 927.0 | 1366.0 | 2.0 |  |
| [(Fe0.7Co0.3)72Mo4B24]94Dy6 | 845.0 | 929.0 | 1367.0 | 2.0 |  |
| [(Fe0.8Co0.2)72Mo4B24]94Dy6 | 852.0 | 944.0 | 1370.0 | 3.0 |  |
| [(Fe0.9Co0.1)72Mo4B24]94Dy6 | 860.0 | 945.0 | 1385.0 | 2.0 |  |
| Gd55Ni22Mn3Al20 | 553.0 | 603.0 | 955.0 | 2.0 |  |
| La62Cu24Al10.5Mg3.5 | 386.0 | 431.0 | 712.0 | 10.0 |  |
| La62Cu24Al10.8Mg3.2 | 387.0 | 421.0 | 710.0 | 10.0 |  |
| La62Cu24Al9.8Mg4.2 | 386.0 | 440.0 | 719.0 | 10.0 |  |
| (La0.3Ce0.7)65Al10Co25 | 416.0 | 436.0 | 778.0 | 9.0 |  |
| (La0.4Ce0.6)65Al10Co25 | 425.0 | 448.0 | 789.0 | 12.0 |  |
| (La0.5Ce0.5)65Al10Co25 | 427.0 | 453.0 | 776.0 | 15.0 |  |
| (La0.6Ce0.4)65Al10Co25 | 437.0 | 467.0 | 835.0 | 20.0 |  |
| (La0.7Ce0.3)65Al10Co25 | 437.0 | 472.0 | 850.0 | 25.0 |  |
| (La0.8Ce0.2)65Al10Co25 | 439.0 | 476.0 | 869.0 | 12.0 |  |
| Mg50Ni30La20 | 453.0 | 510.0 | 841.0 | 1.0 |  |
| Mg57Ni26La17 | 454.0 | 499.0 | 831.0 | 2.0 |  |
| Mg60Ni23.6B0.5La15.9 | 454.0 | 493.0 | 824.0 | 2.0 |  |
| Mg60Ni23.6B2.89La13.51 | 434.0 | 498.0 | 811.0 | 1.0 |  |
| Mg60Ni23.6La16.4 | 450.0 | 490.0 | 819.0 | 2.5 |  |
| Mg60Ni23.6Si0.25La16.15 | 453.0 | 492.0 | 826.0 | 1.0 |  |
| Mg60Ni23.6Si0.5La15.9 | 457.0 | 492.0 | 828.0 | 1.0 |  |
| Mg60Ni23.6Y0.25La16.15 | 456.0 | 491.0 | 829.0 | 3.5 |  |
| Mg60Ni23.6Y0.25Si0.25La15.9 | 456.0 | 492.0 | 824.0 | 2.0 |  |
| Mg60Ni23.6Y0.5La15.9 | 456.0 | 492.0 | 829.0 | 4.0 |  |
| Mg60Ni23.6Y0.5Si0.1La15.8 | 453.0 | 494.0 | 828.0 | 1.5 |  |
| Mg60Ni23.6Y0.5Si0.5La15.75 | 457.0 | 490.0 | 824.0 | 1.0 |  |
| Mg60Ni23.6Y0.75La15.65 | 456.0 | 495.0 | 839.0 | 4.0 |  |
| Mg60Ni23.6Y1La15.4 | 454.0 | 491.0 | 827.0 | 3.0 |  |
| Mg68Ni15Gd10Ag7 | 437.3 | 472.8 | 746.9 | 4.0 |  |
| Mg49Ni15Gd10Ag6 | 439.2 | 474.5 | 756.9 | 7.0 |  |
| Mg69Ni15La16 | 455.0 | 481.0 | 800.0 | 1.0 |  |
| Mg70Ni15Gd10Ag5 | 449.6 | 479.1 | 764.2 | 5.0 |  |
| Mg71Ni15Gd10Ag4 | 440.3 | 485.0 | 773.5 | 4.0 |  |
| Mg73Ni15Gd10Ag2 | 442.3 | 488.7 | 779.8 | 3.5 |  |
| Mg74Ni15Gd10Ag1 | 442.3 | 482.7 | 791.4 | 3.0 |  |
| Mg75Ni15Gd10 | 449.0 | 482.0 | 799.1 | 3.0 |  |
| Ti43.15Zr9.59Cu36.24Ni9.06Sn1.96 | 649.0 | 699.0 | 1167.0 | 3.0 |  |
| Ti44.10Zr9.8Cu37.04Ni7.06Sn2 | 650.0 | 692.0 | 1167.0 | 2.0 |  |
| (Ti0.45Cu0.378Zr0.1Ni0.072)100 | 641.0 | 680.0 | 1167.0 | 1.0 |  |
| (Ti0.45Cu0.378Zr0.1Ni0.072)98Sn2 | 650.0 | 692.0 | 1167.0 | 2.0 |  |
| (Ti0.45Cu0.378Zr0.1Ni0.072)96Sn4 | 666.0 | 715.0 | 1156.0 | 2.0 |  |
| (Ti0.45Cu0.378Zr0.1Ni0.072)94Sn6 | 683.0 | 739.0 | 1169.0 | 1.0 |  |
| Y6Fe72B22 | 898.4 | 948.6 | 1412.6 | 2.0 |  |
| (Y0.06Fe0.72B0.22)98Nb2 | 918.0 | 980.0 | 1411.5 | 4.0 |  |
| (Y0.06Fe0.72B0.22)98Ta2 | 917.3 | 978.8 | 1415.4 | 4.0 |  |
| (Y0.06Fe0.72B0.22)98Ti2 | 914.5 | 973.7 | 1416.0 | 3.0 |  |
| Zr42Au6Cu36Al8Ag8 | 723.0 | 813.0 | 1159.0 | 15.0 |  |
| Zr42Fe6Cu36Al8Ag8 | 708.0 | 797.0 | 1238.0 | 4.0 |  |
| Zr42Hf6Cu36Al8Ag8 | 695.0 | 796.0 | 1187.0 | 15.0 |  |
| Zr42Nb6Cu36Al8Ag8 | 715.0 | 755.0 | 1218.0 | 4.0 |  |
| Zr42Ni6Cu36Al8Ag8 | 695.0 | 779.0 | 1131.0 | 25.0 |  |
| Zr42Pd6Cu36Al8Ag8 | 709.0 | 796.0 | 1155.0 | 20.0 |  |
| Zr42Ti6Cu36Al8Ag8 | 704.0 | 731.0 | 1212.0 | 4.0 |  |
| Zr44Au4Cu36Al8Ag8 | 713.0 | 806.0 | 1153.0 | 20.0 |  |
| Zr44Fe4Cu36Al8Ag8 | 706.0 | 799.0 | 1213.0 | 9.0 |  |
| Zr44Hf4Cu36Al8Ag8 | 694.0 | 795.0 | 1155.0 | 20.0 |  |
| Zr44Nb4Cu36Al8Ag8 | 709.0 | 759.0 | 1209.0 | 9.0 |  |
| Zr44Ni4Cu36Al8Ag8 | 693.0 | 788.0 | 1129.0 | 30.0 |  |
| Zr44Pd4Cu36Al8Ag8 | 705.0 | 795.0 | 1153.0 | 25.0 |  |
| Zr44Ti4Cu36Al8Ag8 | 700.0 | 738.0 | 1181.0 | 9.0 |  |
| Zr46Au2Cu36Al8Ag8 | 705.0 | 799.0 | 1153.0 | 20.0 |  |
| Zr46Cu27.64Ag8.36Al8Be10 | 697.0 | 813.0 | 1129.0 | 35.0 |  |
| Zr46Fe2Cu36Al8Ag8 | 705.0 | 806.0 | 1169.0 | 10.0 |  |
| Zr46Hf2Cu36Al8Ag8 | 692.0 | 794.0 | 1147.0 | 20.0 |  |
| Zr46Nb2Cu36Al8Ag8 | 707.0 | 765.0 | 1150.0 | 10.0 |  |
| Zr46Ni2Cu36Al8Ag8 | 687.0 | 797.0 | 1131.0 | 25.0 |  |
| Zr46Pd2Cu36Al8Ag8 | 699.0 | 794.0 | 1140.0 | 30.0 |  |
| Zr46Ti2Cu36Al8Ag8 | 696.0 | 750.0 | 1145.0 | 10.0 |  |
| Zr48Cu36Al9Ag7 | 710.0 | 776.0 | 1151.0 | 10.4 |  |
| Zr49Ti14Ni20Cu17 | 625.0 | 674.0 | 1095.0 | 4.0 |  |
| Zr49Ti17Ni20Cu14 | 631.0 | 669.0 | 1087.0 | 3.0 |  |
| Zr50.7Cu28Al12.3Ni9 | 719.0 | 780.0 | 1158.0 | 7.0 |  |
| Zr50Ti2Al10Cu38 | 687.0 | 746.0 | 1154.0 | 5.0 |  |
| Zr61.5Al10.7Cu13.65Ni14.15 | 669.9 | 737.5 | 1154.6 | 5.5 | ^20^ |
| Zr62.5Al12.1Cu7.95Ni17.45 | 672.3 | 743.8 | 1172.2 | 7.5 |  |
| Zr63.5Al10.7Cu10.7Ni15.1 | 658.4 | 729.9 | 1165.8 | 6.0 |  |
| Zr63Al11.4Cu9.3Ni16.3 | 663.3 | 732.2 | 1164.3 | 6.5 |  |
| Zr64Al10.1Cu11.7Ni14.2 | 657.9 | 717.3 | 1158.5 | 5.0 |  |
| Zr65Al8.7Cu14.4Ni11.9 | 647.0 | 709.5 | 1164.8 | 4.0 |  |
| Zr70Al8Cu19Ni3 | 630.0 | 689.0 | 1200.0 | 1.5 |  |
| Zr70Al8Cu3Ni19 | 640.0 | 710.0 | 1220.0 | 1.5 |  |
| (Zr0.5Cu0.38Ti0.02Al0.10)95Y5 | 664.0 | 721.0 | 1145.0 | 6.0 |  |
| (Zr0.5Cu0.38Ti0.02Al0.10)97Y3 | 665.0 | 736.0 | 1146.0 | 16.0 |  |
| (Zr0.5Cu0.38Ti0.02Al0.10)98Y2 | 671.0 | 739.0 | 1148.0 | 20.0 |  |
| (Zr0.5Cu0.38Ti0.02Al0.10)99Y1 | 681.0 | 741.0 | 1145.0 | 18.0 |  |
| Ti55Zr15Be20Ni10 | 604.0 | 639.0 | 1171.0 | 5.0 | ^21^ |
| (Ti55Zr15Be20Ni10)98Cu2 | 603.0 | 642.0 | 1143.0 | 7.0 |  |
| (Ti55Zr15Be20Ni10)96Cu4 | 599.0 | 646.0 | 1123.0 | 10.0 |  |
| (Ti55Zr15Be20Ni10)94Cu6 | 593.0 | 649.0 | 1079.0 | 8.0 |  |
| (Ti55Zr15Be20Ni10)92Cu8 | 586.0 | 653.0 | 1050.0 | 8.0 |  |
| (Ti55Zr15Be20Ni10)90Cu10 | 582.0 | 656.0 | 1046.0 | 7.0 |  |
| Zr56.25Al18.75Co25 | 761.0 | 811.0 | 1361.0 | 3.0 | ^22^ |
| Zr56.25Al18.75(Co0.875Cu0.125)25 | 754.0 | 811.0 | 1284.0 | 8.0 |  |
| Zr56.25Al18.75(Co0.75Cu25)25 | 749.0 | 810.0 | 1277.0 | 10.0 |  |
| Zr56.25Al18.75(Co0.625Cu0.375)25 | 741.0 | 807.0 | 1270.0 | 8.0 |  |
| Zr56.25Al18.75(Co0.5Cu0.5)25 | 735.0 | 798.0 | 1242.0 | 5.0 |  |
| Cu50Zr43Al7 | 717.0 | 784.0 | 1205.0 | 10.0 | ^23^ |
| (Cu50Zr43Al7)98Er2 | 697.0 | 773.0 | 1172.0 | 15.0 |  |
| (Cu50Zr43Al7)96Er4 | 687.0 | 751.0 | 1154.0 | 12.0 |  |
| (Cu50Zr43Al7)94Er6 | 653.0 | 700.0 | 1150.0 | 10.0 |  |
| Zr63.5Al9Fe4.5Cu23 | 658.0 | 768.0 | 1186.0 | 6.0 | ^24^ |
| Zr62Hf1.5Al9Fe4.5Cu23 | 658.0 | 755.0 | 1202.0 | 8.0 |  |
| Zr62Hf3Al9Fe4.5Cu23 | 659.0 | 752.0 | 1209.0 | 10.0 |  |
| Zr59Hf4.5Al9Fe4.5Cu23 | 668.0 | 770.0 | 1222.0 | 8.0 |  |
| Zr57.5Hf6Al9Fe4.5Cu23 | 668.0 | 752.0 | 1229.0 | 6.0 |  |
| Zr48Cu36Al12Ag4 | 705.0 | 746.0 | 1109.0 | 3.0 | ^25^ |
| Zr48Cu36Al10Ag6 | 699.0 | 753.0 | 1153.0 | 7.0 |  |
| Zr48Cu36Al8Ag8 | 688.0 | 771.0 | 1126.0 | 12.0 |  |
| Zr48Cu36Al6Ag10 | 689.0 | 755.0 | 1099.0 | 10.0 |  |
| Zr48Cu36Al4Ag12 | 669.0 | 746.0 | 1108.0 | 6.0 |  |
| Zr48Cu36Al2Ag14 | 662.0 | 727.0 | 1118.0 | 2.0 |  |
| Ti40Zr10Cu34Pd14Ga2 | 667.0 | 711.0 | 1202.0 | 3.0 | ^26^ |
| Ti40Zr10Cu32Pd14Ga4 | 670.0 | 717.0 | 1201.0 | 3.0 |  |
| Ti40Zr10Cu28Pd14Ga8 | 680.0 | 726.0 | 1185.0 | 2.0 |  |
| Ti40Zr10Cu26Pd14Ga10 | 710.0 | 752.0 | 1191.0 | 2.0 |  |
| Au51.6Ag5.8Pd2.4Cu20.2Ga6.7Si13.3 | 376.0 | 428.0 | 679.0 | 3.0 | ^27^ |
| Au52.6Ag5.8Pd2.4Cu18.7Ga8.2Si12.3 | 372.0 | 426.0 | 668.0 | 2.0 |  |
| Mg68Zn28Ca4 | 359.0 | 378.0 | 665.0 | 3.5 | ^28^ |
| (Mg68Zn28Ca4)99.5Ga0.5 | 358.0 | 372.0 | 703.0 | 2.0 |  |
| (Mg68Zn28Ca4)99.25Ga0.75 | 359.0 | 378.0 | 677.0 | 3.5 |  |
| (Mg68Zn28Ca4)99Ga1 | 358.0 | 378.0 | 664.0 | 5.0 |  |
| (Mg68Zn28Ca4)98.75Ga1.25 | 358.0 | 376.0 | 668.0 | 3.5 |  |
| Ce70Ga4Cu26 | 360.0 | 396.0 | 709.0 | 3.0 | ^29^ |
| Ce70Ga6Cu24 | 362.0 | 418.0 | 696.0 | 10.0 |  |
| Ce70Ga10Cu20 | 365.0 | 441.0 | 681.0 | 10.0 |  |
| Ce70Ga12Cu18 | 387.0 | 428.0 | 664.0 | 8.0 |  |
| Ce70Ga15Cu15 | 394.0 | 428.0 | 664.0 | 3.0 |  |
| Ce70Ga8.5Cu20Ni1.5 | 360.0 | 436.0 | 657.0 | 14.0 |  |
| Ce70Ga8.5Cu18.5Ni3 | 361.0 | 416.0 | 653.0 | 10.0 |  |
| Ce70Ga8.5Cu16.5Ni5 | 367.0 | 406.0 | 654.0 | 6.0 |  |
| Ce70Ga8.5Cu13Ni8.5 | 371.0 | 397.0 | 662.0 | 3.0 |  |
| Ce70Ga8.5Cu11Ni10.5 | 379.0 | 401.0 | 672.0 | 2.0 |  |
| Ce70Al10Cu20 | 357.0 | 421.0 | 660.0 | 2.0 |  |
| Ce68Al10Cu20Ni2 | 366.0 | 454.0 | 676.0 | 5.0 |  |
| Fe59Cr6Mo14C15B6 | 806.0 | 858.0 | 1436.0 | 1.5 | ^30^ |
| Fe48Cr15Mo14C15B6Er2 | 843.0 | 893.0 | 1443.0 | 12.0 |  |
| Fe47Cr20Mo10C15B6Y2 | 854.0 | 909.0 | 1494.0 | 5.0 |  |
| Fe45Cr20Mo10W2C15B6Y2 | 856.0 | 913.0 | 1500.0 | 6.0 |  |
| Fe43Cr20Mo10W4C15B6Y2 | 858.0 | 920.0 | 1503.0 | 8.0 |  |
| Fe41Cr20Mo10W6C15B6Y2 | 881.0 | 918.0 | 1518.0 | 6.0 |  |
| Fe40Ni35P10C10B5 | 643.0 | 698.0 | 1220.0 | 1.5 | ^31^ |
| Fe40Co35P10C10B5 | 704.0 | 760.0 | 1267.0 | 2.0 |  |
| Fe40Co29Ni15P10C10B5 | 675.0 | 735.0 | 1255.0 | 2.5 |  |
| Fe70Cr7Mo3P12C4B4 | 728.0 | 770.0 | 1349.0 | 1.5 | ^32^ |
| Fe62Ni8Cr7Mo3P12C4B4 | 705.0 | 756.0 | 1323.0 | 2.0 |  |
| Fe55Ni15Cr7Mo3P12C4B4 | 690.0 | 750.0 | 1302.0 | 2.5 |  |
| Fe50Ni20Cr7Mo3P12C4B4 | 684.0 | 742.0 | 1299.0 | 2.0 |  |
| Zr56Cu7.5Ni17.5Al15Nb4 | 704.0 | 766.0 | 1280.0 | 6.0 | ^33^ |
| Zr54Cu9.5Ni17.5Al15Nb4 | 711.0 | 761.0 | 1268.0 | 6.0 |  |
| Zr52Cu11.5Ni17.5Al15Nb4 | 716.0 | 769.0 | 1270.0 | 8.0 |  |
| Zr50Cu13.5Ni17.5Al15Nb4 | 720.0 | 765.0 | 1279.0 | 6.0 |  |
| Zr48Cu15.5Ni17.5Al15Nb4 | 729.0 | 771.0 | 1304.0 | 6.0 |  |
| Fe42Co6Cr15Mo14C9B12Tm2 | 876.0 | 925.0 | 1427.0 | 2.0 | ^34^ |
| Fe42Co6Cr15Mo14C11B10Tm2 | 872.0 | 923.0 | 1440.0 | 5.0 |  |
| Fe42Co6Cr15Mo14C13B8Tm2 | 871.0 | 908.0 | 1444.0 | 8.0 |  |
| Fe42Co6Cr15Mo14C15B6Tm2 | 850.0 | 893.0 | 1452.0 | 12.0 |  |
| Fe42Co6Cr15Mo14C17B4Tm2 | 845.0 | 880.0 | 1468.0 | 6.0 |  |
| Zr51Co33Al16 | 775.0 | 828.0 | 1258.0 | 2.0 | ^35^ |
| Zr52Co32Al16 | 770.0 | 824.0 | 1256.0 | 3.0 |  |
| Zr53Co31Al16 | 763.0 | 812.0 | 1257.0 | 5.0 |  |
| Zr54Co30Al16 | 757.0 | 806.0 | 1258.0 | 6.0 |  |
| Zr55Co29Al16 | 750.0 | 799.0 | 1258.0 | 7.0 |  |
| Zr56Co28Al16 | 743.0 | 792.0 | 1258.0 | 7.0 |  |
| Zr56Co27Al16 | 735.0 | 787.0 | 1260.0 | 5.0 |  |
| Zr58Co28Al16 | 729.0 | 780.0 | 1282.0 | 2.0 |  |
| Zr62Ti1.5Al9Fe4.5Cu23 | 662.0 | 740.0 | 1185.0 | 6.0 | ^36^ |
| Zr60.5Ti3Al9Fe4.5Cu23 | 662.0 | 732.0 | 1165.0 | 10.0 |  |
| Zr59Ti4.5Al9Fe4.5Cu23 | 662.0 | 716.0 | 1153.0 | 8.0 |  |
| Zr57.5Ti6Al9Fe4.5Cu23 | 661.0 | 705.0 | 1149.0 | 3.0 |  |
| Zr31Ti27Be26Cu10Fe6 | 617.0 | 685.0 | 1037.0 | 13.0 | ^37^ |
| Zr31Ti27Be26Cu10Al6 | 607.0 | 678.0 | 1060.0 | 13.0 |  |
| Zr31Ti27Be26Cu10Ag6 | 620.0 | 675.0 | 1057.0 | 14.0 |  |
| Zr31Ti27Be26Cu10Ni6 | 610.0 | 682.0 | 1023.0 | 15.0 |  |
| Zr31Ti27Be26Cu10Cr6 | 611.0 | 678.0 | 1018.0 | 14.0 |  |
| Zr31Ti27Be26Cu10V6 | 610.0 | 666.0 | 1027.0 | 15.0 |  |
| Zr28Ti24Be23Cu9Ni10Fe6 | 643.0 | 694.0 | 1107.0 | 4.0 |  |
| Zr28Ti24Be23Cu9Ni10Al6 | 642.0 | 685.0 | 1087.0 | 9.0 |  |
| Zr28Ti24Be23Cu9Ni10Ag6 | 633.0 | 682.0 | 1074.0 | 14.0 |  |
| Zr28Ti24Be23Cu9Ni10Cr6 | 637.0 | 686.0 | 1071.0 | 6.0 |  |
| Zr28Ti24Be23Cu9Ni10V6 | 635.0 | 677.0 | 1064.0 | 7.0 |  |
| La62Al14Ni24 | 431.0 | 451.0 | 980.0 | 2.0 | ^38^ |
| La62Al14(Ni0.9Co0.1)24 | 452.0 | 471.0 | 983.0 | 8.0 |  |
| La62Al14(Ni0.8Co0.2)24 | 434.0 | 456.0 | 986.0 | 20.0 |  |
| La62Al14(Ni0.7Co0.3)24 | 406.0 | 427.0 | 991.0 | 7.0 |  |
| (Co0.8Fe0.2)71.5Y3.5B25 | 827.0 | 870.0 | 1458.0 | 1.0 | ^39^ |
| (Co0.7Fe0.3)71.5Y3.5B25 | 826.0 | 876.0 | 1438.0 | 2.0 |  |
| (Co0.6Fe0.4)71.5Y3.5B25 | 824.0 | 884.0 | 1468.0 | 1.0 |  |
| (Co0.5Fe0.5)71.5Y3.5B25 | 829.0 | 893.0 | 1493.0 | 1.0 |  |
| (Co0.7Fe0.3)72Y3B25 | 822.0 | 870.0 | 1481.0 | 1.0 |  |
| (Co0.7Fe0.3)71Y4B25 | 827.0 | 878.0 | 1447.0 | 1.5 |  |
| (Co0.7Fe0.3)70Y5B25 | 841.0 | 888.0 | 1470.0 | 1.5 |  |
| (Fe71.2B24Y4.8)96Nb4 | 840.0 | 903.0 | 1391.0 | 3.0 | ^40^ |
| (Fe71.2B24Y4.8)96Nb3Mo1 | 846.0 | 910.0 | 1399.0 | 5.0 |  |
| (Fe71.2B24Y4.8)96Nb2Mo2 | 858.0 | 927.0 | 1412.0 | 6.0 |  |
| (Fe71.2B24Y4.8)96Nb1Mo3 | 845.0 | 911.0 | 1415.0 | 5.0 |  |
| (Ti41Zr25Be28Fe6)98Cu2 | 613.0 | 720.0 | 1128.0 | 12.0 | ^41^ |
| (Ti41Zr25Be28Fe6)95Cu5 | 617.0 | 714.0 | 1110.0 | 12.0 |  |
| (Ti41Zr25Be28Fe6)85Cu15 | 622.0 | 682.0 | 1116.0 | 15.0 |  |
| (Ti41Zr25Be28Fe6)80Cu20 | 627.0 | 687.0 | 1114.0 | 7.0 |  |
| Cu48Zr48Al4 | 683.0 | 743.0 | 1191.0 | 2.0 | ^42^ |
| Cu45Zr48Al4Ga3 | 703.0 | 763.0 | 1174.0 | 5.0 |  |
| Cu40Zr48Al4Ag8 | 684.0 | 756.0 | 1129.0 | 9.0 |  |
| Cu46.5Zr48Al4Nb1.5 | 687.0 | 744.0 | 1181.0 | 5.0 |  |
| Cu47.5Zr48Al4Ni0.5 | 688.0 | 743.0 | 1193.0 | 3.0 |  |
| Cu47.5Zr48Al4V0.5 | 695.0 | 747.0 | 1192.0 | 2.0 |  |
| Cu47.7Zr48Al4Co0.3 | 690.0 | 744.0 | 1195.0 | 3.0 |  |
| Cu47.5Zr48Al4Cr0.5 | 689.0 | 744.0 | 1195.0 | 3.0 |  |
| Cu47.5Zr48Al4Fe0.5 | 692.0 | 743.0 | 1194.0 | 3.0 |  |
| Zr20Ti20Hf20Cu20Be20 | 632.0 | 715.0 | 1194.0 | 5.0 | ^43^ |
| Zr20Ti20Hf20Ni20Be20 | 657.0 | 709.0 | 1108.0 | 8.0 |  |
| Zr20Ti20Hf20Co20Be20 | 683.0 | 722.0 | 1187.0 | 3.0 |  |
| Ti20Zr20Hf20Be20Cu20 | 630.0 | 708.0 | 1164.0 | 12.0 | ^44^ |
| Ti20Zr20Hf20Be20Cu17.5Ni2.5 | 641.0 | 693.0 | 1156.0 | 12.0 |  |
| Ti20Zr20Hf20Be20Cu15Ni5 | 638.0 | 694.0 | 1102.0 | 15.0 |  |
| Ti20Zr20Hf20Be20Cu12.5Ni7.5 | 644.0 | 692.0 | 1090.0 | 20.0 |  |
| Ti20Zr20Hf20Be20Cu10Ni10 | 643.0 | 695.0 | 1066.0 | 25.0 |  |
| Ti20Zr20Hf20Be20Cu7.5Ni12.5 | 632.0 | 684.0 | 1040.0 | 30.0 |  |
| Ti20Zr20Hf20Be20Cu5Ni15 | 644.0 | 696.0 | 1109.0 | 20.0 |  |
| Ti20Zr20Hf20Be20Ni20 | 646.0 | 701.0 | 1108.0 | 15.0 |  |

References:

1 Dai, C.-L. *et al.* A new centimeter–diameter Cu-based bulk metallic glass. *Scripta materialia* **54**, 1403-1408 (2006).

2 Zhang, Q., Zhang, H., Deng, Y., Ding, B. & Hu, Z. Bulk metallic glass formation of Cu–Zr–Ti–Sn alloys. *Scripta materialia* **49**, 273-278 (2003).

3 Long, Z. *et al.* A new criterion for predicting the glass-forming ability of bulk metallic glasses. *Journal of alloys and compounds* **475**, 207-219 (2009).

4 Zhang, Q., Zhang, W. & Inoue, A. New Cu–Zr-based bulk metallic glasses with large diameters of up to 1.5 cm. *Scripta materialia* **55**, 711-713 (2006).

5 Zhang, W., Zhang, Q., Qin, C. & Inoue, A. Formation and properties of new Cu-based bulk glassy alloys with critical diameters up to 1.5 cm. *Journal of Materials Research* **24**, 2935-2940 (2009).

6 Kim, Y., Lee, J., Cha, P., Ahn, J. & Fleury, E. Enhanced glass forming ability and mechanical properties of new Cu-based bulk metallic glasses. *Materials Science and Engineering: A* **437**, 248-253 (2006).

7 Zhang, W., Jia, F., Zhang, Q. & Inoue, A. Effects of additional Ag on the thermal stability and glass-forming ability of Cu–Zr binary glassy alloys. *Materials Science and Engineering: A* **459**, 330-336 (2007).

8 Zhang, Q., Zhang, W. & Inoue, A. Fabrication of new Cu34Pd2Zr48Ag8Al8 bulk glassy alloy with a diameter of 30 mm. *Materials transactions* **48**, 3031-3033 (2007).

9 Nagy, E., Rontó, V., Sólyom, J. & Roósz, A. in *Journal of Physics: Conference Series.* 012035 (IOP Publishing).

10 Zhang, B., Zhao, D., Pan, M., Wang, R. & Wang, W. Formation of cerium-based bulk metallic glasses. *Acta Materialia* **54**, 3025-3032 (2006).

11 Liu, Z., Chan, K. C. & Liu, L. Enhanced glass forming ability and plasticity of a Ni-free Zr-based bulk metallic glass. *Journal of Alloys and Compounds* **487**, 152-156 (2009).

12 Zhu, J. *et al.* Formation of Zr-based bulk metallic glass with large amount of yttrium addition. *Intermetallics* **92**, 55-61 (2018).

13 Li, Y. *et al.* Unusual compressive plasticity of a centimeter-diameter Zr-based bulk metallic glass with high Zr content. *Journal of Alloys and Compounds* **504**, S2-S5 (2010).

14 Hua, N. *et al.* Formation and mechanical properties of Ni-free Zr-based bulk metallic glasses. *Journal of Alloys and Compounds* **509**, S175-S178 (2011).

15 Li, Y. *et al.* Enhancement of glass-forming ability and corrosion resistance of Zr-based Zr-Ni-Al bulk metallic glasses with minor addition of Nb. *Journal of Applied Physics* **110**, 023513 (2011).

16 Hua, N. *et al.* Ni-and Cu-free Zr–Al–Co–Ag bulk metallic glasses with superior glass-forming ability. *Journal of Materials Research* **26**, 539-546 (2011).

17 Zhang, C. *et al.* Enhancement of glass-forming ability and bio-corrosion resistance of Zr–Co–Al bulk metallic glasses by the addition of Ag. *Journal of Alloys and Compounds* **504**, S163-S167 (2010).

18 Chen, Q., Liu, L. & Zhang, S.-M. The potential of Zr-based bulk metallic glasses as biomaterials. *Frontiers of Materials Science in China* **4**, 34-44 (2010).

19 Long, Z. *et al.* A new correlation between the characteristics temperature and glass-forming ability for bulk metallic glasses. *Journal of Thermal Analysis and Calorimetry* **132**, 1645-1660 (2018).

20 Tripathi, M. K., Ganguly, S., Dey, P. & Chattopadhyay, P. Evolution of glass forming ability indicator by genetic programming. *Computational Materials Science* **118**, 56-65 (2016).

21 Gu, J.-L. *et al.* Effects of Cu addition on the glass forming ability and corrosion resistance of Ti-Zr-Be-Ni alloys. *Journal of Alloys and Compounds* **725**, 573-579 (2017).

22 Han, K., Qiang, J., Wang, Y. & Häussler, P. Zr-Al-Co-Cu bulk metallic glasses for biomedical devices applications. *Journal of Alloys and Compounds* **729**, 144-149 (2017).

23 Malekan, M., Rashidi, R. & Shabestari, S. G. Mechanical properties and crystallization kinetics of Er-containing Cu–Zr–Al bulk metallic glasses with excellent glass forming ability. *Vacuum* **174**, 109223 (2020).

24 Han, K., Wang, Y., Qiang, J., Jiang, H. & Gu, L. Low-cost Zr-based bulk metallic glasses for biomedical devices applications. *Journal of Non-Crystalline Solids* **520**, 119442 (2019).

25 Błyskun, P., Kowalczyk, M., Cieślak, G. & Kulik, T. Glass forming ability of Zr48Cu36Al16-xAgx alloys determined by three different methods. *Journal of Non-Crystalline Solids* **515**, 106-112 (2019).

26 Bera, S. *et al.* Tuning the glass forming ability and mechanical properties of Ti-based bulk metallic glasses by Ga additions. *Journal of Alloys and Compounds* **793**, 552-563 (2019).

27 Neuber, N. *et al.* The role of Ga addition on the thermodynamics, kinetics, and tarnishing properties of the Au-Ag-Pd-Cu-Si bulk metallic glass forming system. *Acta Materialia* **165**, 315-326 (2019).

28 Zai, W., Man, H. C., Su, Y., Li, G. & Lian, J. Impact of microalloying element Ga on the glass-forming ability (GFA), mechanical properties and corrosion behavior of Mg–Zn–Ca bulk metallic glass. *Materials Chemistry and Physics* **255**, 123555 (2020).

29 Zhao, Y. & Zhang, B. Evaluating the correlation between liquid fragility and glass-forming ability in the extremely strong Ce-based bulk metallic glasses. *Journal of Applied Physics* **122**, 115107 (2017).

30 Liang, D.-d. *et al.* Effect of W addition on the glass forming ability and mechanical properties of Fe-based metallic glass. *Journal of Alloys and Compounds* **731**, 1146-1150 (2018).

31 Zhang, W. *et al.* Glass-forming ability and thermoplastic formability of ferromagnetic (Fe, Co, Ni) 75P10C10B5 metallic glasses. *Journal of Alloys and Compounds* **707**, 57-62 (2017).

32 Wang, S., Li, Y., Wang, X., Yamaura, S. & Zhang, W. Glass-forming ability, thermal properties, and corrosion resistance of Fe-based (Fe, Ni, Mo, Cr)-PCB metallic glasses. *Journal of Non-Crystalline Solids* **476**, 75-80 (2017).

33 Deng, X. *et al.* Excellent room-temperature mechanical properties in the high glass-forming Zr− Cu− Ni− Al− Nb alloy system. *Materials Research Express* **6**, 086551 (2019).

34 Wang, Q. *et al.* Effects of C/B ratio on glass-forming ability and low-temperature magnetic behavior of FeCoCrMoCBTm metallic glass. *Journal of Alloys and Compounds* **864**, 158211 (2021).

35 Yu, K. *et al.* Glass forming ability and bending plasticity evolutions in Zr-Co-Al bulk metallic glasses and their structural origin. *Journal of Non-Crystalline Solids* **488**, 52-62 (2018).

36 Han, K.-M., Jiang, H., Wang, Y.-M. & Qiang, J.-B. Zr–Ti–Al–Fe–Cu bulk metallic glasses for biomedical device application. *Rare Metals* **40**, 1239-1246 (2021).

37 Bizhanova, G., Li, F., Ma, Y., Gong, P. & Wang, X. Development and crystallization kinetics of novel near-equiatomic high-entropy bulk metallic glasses. *Journal of Alloys and Compounds* **779**, 474-486 (2019).

38 Yang, G., Lian, J., Wang, R. & Wu, N. Similar atom substitution effect on the glass forming ability in (LaCe) Al-(NiCo) bulk metallic glasses using electron structure guiding. *Journal of Alloys and Compounds* **786**, 250-256 (2019).

39 Liang, X. *et al.* Roles of Y and Fe contents on glass-forming ability, thermal stability, and magnetic properties of Co-based Co–Fe–Y–B bulk metallic glasses. *Intermetallics* **132**, 107135 (2021).

40 Zhu, K.-r., Jiang, W., Wu, J.-l. & Zhang, B. Effect of Mo on properties of the industrial Fe–B-alloy-derived Fe-based bulk metallic glasses. *International Journal of Minerals, Metallurgy, and Materials* **24**, 926-930 (2017).

41 Gong, P. *et al.* A Ti–Zr–Be–Fe–Cu bulk metallic glass with superior glass-forming ability and high specific strength. *Intermetallics* **43**, 177-181 (2013).

42 Cao, D. *et al.* Enhancement of glass-forming ability and plasticity via alloying the elements having positive heat of mixing with Cu in Cu48Zr48Al4 bulk metallic glass. *Journal of Alloys and Compounds* **777**, 382-391 (2019).

43 Zong, H. *et al.* Excellent glass forming ability and plasticity in high entropy Zr20Ti20Hf20M20Be20 (M= Cu, Ni, Co) alloys. *Results in physics* **8**, 253-256 (2018).

44 Zhao, S. *et al.* Pseudo-quinary Ti20Zr20Hf20Be20 (Cu20-xNix) high entropy bulk metallic glasses with large glass forming ability. *Materials & Design* **87**, 625-631 (2015).
